# Cost-Effectiveness of Price Subsidies on Fortified Packaged Infant Cereals in Reducing Iron Deficiency Anemia in 6-23-Month-Old-Children in Urban India

**DOI:** 10.1371/journal.pone.0152800

**Published:** 2016-04-13

**Authors:** Rafael Plessow, Narendra Kumar Arora, Beatrice Brunner, Simon Wieser

**Affiliations:** 1 Winterthur Institute of Health Economics, Zurich University of Applied Sciences, Winterthur, Switzerland; 2 International Clinical Epidemiology Network, INCLEN, New Delhi, India; University College London, UNITED KINGDOM

## Abstract

**Introduction:**

Iron deficiency anaemia (IDA) is a major public health problem in India and especially harmful in early childhood due to its impact on cognitive development and increased all-cause mortality. We estimate the cost-effectiveness of price subsidies on fortified packaged infant cereals (F-PICs) in reducing IDA in 6-23-monthold children in urban India.

**Materials and Methods:**

Cost-effectiveness is estimated by comparing the net social cost of price subsidies with the disability-adjusted life-years (DALYs) averted with price subsidies. The net social costs correspond to the cost of the subsidy minus the monetary costs saved by reducing IDA. The estimation proceeds in three steps: 1) the current lifetime costs of IDA are assessed with a health economic model combining the prevalence of anemia, derived from a large population survey, with information on the health consequences of IDA and their costs in terms of mortality, morbidity, and DALYs. 2) The effects of price subsidies on the demand for F-PICs are assessed with a market survey among 4801 households in 12 large Indian cities. 3) The cost-effectiveness is calculated by combining the findings of the first two steps with the results of a systematic review on the effectiveness of F-PICs in reducing IDA. We compare the cost-effectiveness of interventions that differ in the level of the subsidy and in the socio-economic strata (SES) eligible for the subsidy.

**Results:**

The lifetime social costs of IDA in 6-23-month-old children in large Indian cities amount to production losses of 3222 USD and to 726,000 DALYs. Poor households incur the highest costs, yet even wealthier households suffer substantial losses. The market survey reveals that few households currently buy F-PICs, with the share ranging from 14% to 36%. Wealthier households are generally more likely to buy FPICs. The costs of the subsidies per DALY averted range from 909 to 3649 USD. Interventions targeted at poorer households are most effective. Almost all interventions are cost saving from a societal perspective when taking into account the reduction of future production losses. Return per DALY averted ranges between gains of 1655 USD to a cost of 411 USD.

**Conclusion:**

Price subsidies on F-PICs are a cost-effective way to reduce the social costs of IDA in 6-23-month-old children in large Indian cities. Interventions targeting poorer households are especially cost-effective.

## Introduction

Inadequate nutrition has a severe impact on health in India. The single most important nutritional risk factor in India is iron deficiency, accounting for more than 3% of all disability-adjusted life-years (DALYs) according to the WHO Global Burden of Disease project [1]. Iron deficiency anemia (IDA) is highly prevalent among Indian children in spite of substantial economic growth and numerous programs aimed at the reduction of anemia [2]. Iron deficiency in early childhood is especially detrimental due to increased mortality and its permanent impact on cognitive development, which leads to an irreversible loss of productivity in adult life [3].

WHO and UNICEF [4] have provided guidelines on infant and young child feeding which aim to maximize exclusive—and later to continue partial—breast feeding, with the introduction of appropriate complementary feeding after the sixth month. Complementary feeding is important, as children in this age group are in a period of rapid mental and physical development with a particularly high need for micronutrient intake. Their iron stores are slowly depleted, yet their diet provides them with insufficient iron in this period of fast development [5, 6]. Complementary foods thus need to be far more nutrient rich than the foods typically consumed by the households [7].Furthermore, even in countries with a diet rich in animal source foods sufficient iron intake by 6-12-month-old children has probably only been achieved with the widespread use of iron-fortified infant foods [8].

In India IDA has been recognized as a serious public health problem since 1970, when the first anemia prevention program was introduced. The National Nutritional Anemia Control Program, as it is called today, is supposed to cover all children aged 12–59 months with iron supplements. Actual coverage is however at 3.8% only [6]. Targeted interventions are needed to reach this vulnerable group, out of reach of many large interventions, such as staple food fortification.

In a recent study we show that IDA in 6-59-month-old children leads to substantial social costs in India [9]. These costs are mainly due to the effect of IDA in 6-23-month-old children which lead to impaired cognitive development and thus to lower incomes in adult life. We also find that previous calculations have considerably underestimated the DALYs due to IDA.

Interventions with fortified packaged infant cereals (F-PICs) have a number of advantages over other strategies addressing IDA in 6-23-month-old children, such as iron supplementation, iron fortification of staple foods and home fortification. Fortification of staple foods cannot provide sufficient nutrients to small children because, in order to remain safe for the whole population, fortification levels have to be set so low that the limited quantity consumed by 6-23-month-old children does not contain sufficient iron. Iron supplementation is well suited for targeting specific populations, however, compliance is usually low. In India coverage rates are still very low after decades of iron supplementation [2, 10, 11]. Home fortification of complementary foods is likely to be affected by the same issues of compliance. Two recent reports by the Global Alliance for Improved Nutrition (gain) emphasize the importance of convenience, speed of preparation and taste for the uptake of complementary infant foods [12, 13].

This paper estimates the cost-effectiveness of price subsidies on F-PICs in reducing the social costs of IDA in 6-23-month-old children in large Indian cites. We choose packaged infant cereals as the food to be iron-fortified because they are well suited to deliver iron to young children in complementary feeding alongside breastfeeding. By combining macro and micronutrients, fortified packaged foods are safer than home fortification. Due to the macronutrients included, the quantity of F-PIC a child may eat per day is limited. F-PICs are also exclusively targeted at young children and unlikely to be consumed by other members of the household. Furthermore, the packaging of F-PICs assures an appropriate conservation of the nutritional content. In addition, we focus on big cities with a population of over 500,000, which in 2011 included a population of over 146 million [14], because F-PICs are readily available there. Hence, price subsidies may be implemented with a market based approach using the existing commercial distribution channels.

## Methods

### Overview of study design

**Fig 1:**
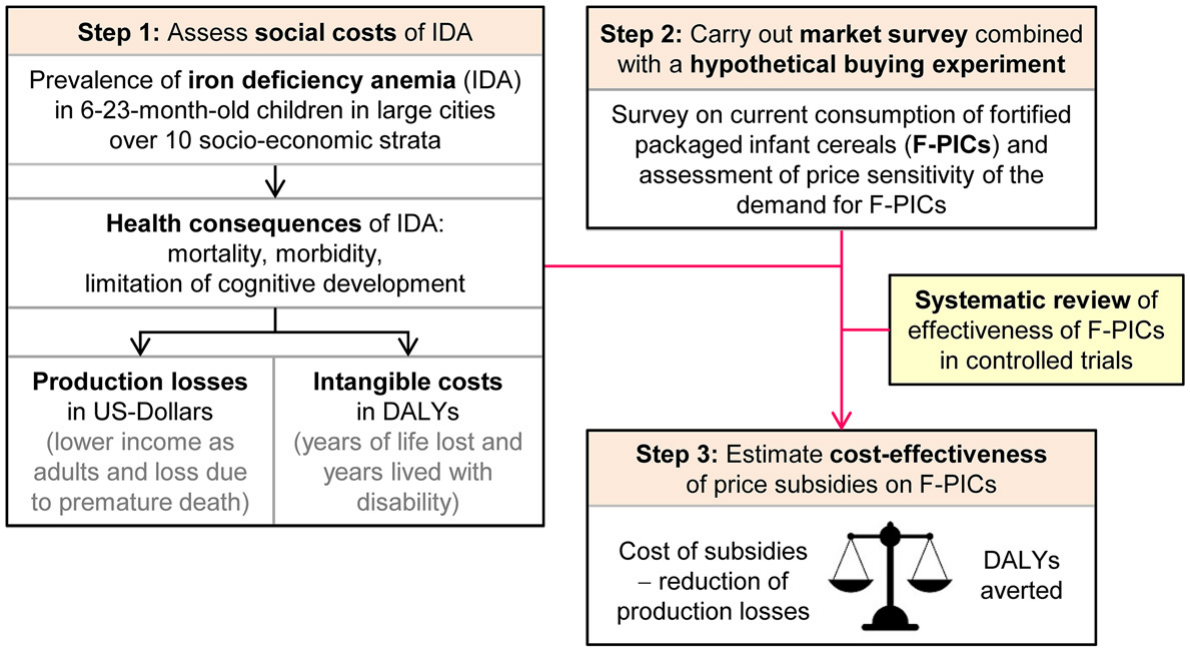
Overview of health economic model. The health economic model assesses the cost-effectiveness of hypothetical price subsidies on F-PICs by proceeding in three steps: Step 1) The social costs of IDA in 6-23-old-children are assessed by linking the prevalence of IDA with its health and cost consequences. Step 2) A market survey is carried out to assess current consumption of F-PICs and to assess the price sensitivity of demand for F-PICs with a hypothetical buying experiment. Step 3) The cost-effectiveness of price subsidies on F-PICs is assessed by combining the results of the first two steps with the results of a systematic review on the effectiveness of F-PICs in reducing anemia in 6-23-month-old children.

This study evaluates the cost-effectiveness of interventions with price subsidies on F-PICs in reducing IDA in 6-23-month-old children in large Indian cities. The study proceeds in three steps (Fig 1): *First*, we assess the social costs of IDA in 6-23-month-old children in large Indian cities by adapting the model developed in Plessow et al. [9]. *Second*, we carry out a survey on the current consumption of F-PICs and a hypothetical buying experiment to assess the price sensitivity of demand for F-PICs. *Third*, we combine the results of the first two steps with the results of a systematic review on the effectiveness of F-PICs in reducing anemia in young children [15]. This allows us to estimate the cost-effectiveness of price subsidies on F-PICs in reducing the social cost of IDA. The cost-effectiveness is estimated by comparing the net social cost of price subsidies with the DALYs averted by the intervention. The net social costs correspond to the cost of the subsidy minus the production losses averted by reducing IDA.

### Social costs of IDA in large Indian cities

We first calculate the social costs due to IDA in 6-23-month-old children in large Indian cities, using our health economic model of the lifetime cost-consequences of IDA in India [9]. We adapt the model for large Indian cities by adjusting the population size, IDA prevalence and mortality rates for large cities with more than 500,000 inhabitants. IDA prevalence is based on the National Family Health Survey NFHS-3 [16] and assessed by 3 degrees of severity (mild, moderate, severe). The population is stratified over 10 socio-economic strata (SES) determined by the deciles of a wealth index calculated according to DHS methodology [17]. Based on a recent systematic review (14) and a WHO report (15), we attribute 60% of cases of anemia in 6-23-month-old children to iron deficiency.

Costs considered include both intangible costs in terms of DALYs due to mortality and morbidity, and production losses (in monetary terms) due to premature death and lower future income. Costs are calculated separately for each SES.

### Market survey and hypothetical buying experiment

**Fig 2:**
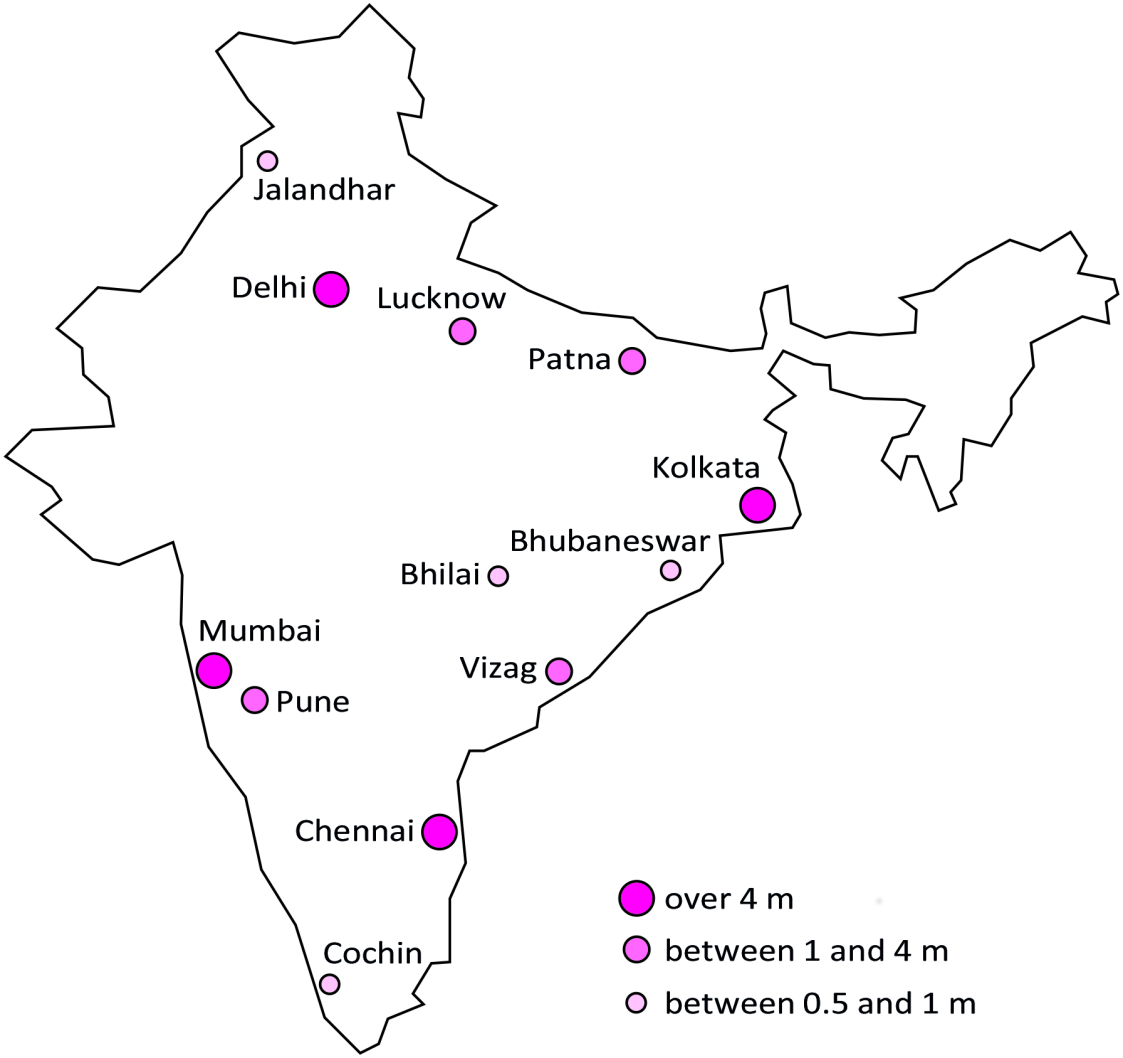
The 12 large Indian cities included in survey by city size category. Total population in Indian cities over 0.5m inhabitants was 146m in 2011 [14], with 58m in cities with more than 4m, 58m in cities between 1 and 4m, and 30m in cities between 0.5 and 1m. Made with Natural Earth. Free vector and raster map data @ naturalearthdata.com.

In a second step we conducted a market survey to assess the current consumption of packaged infant cereals (PIC) as well as the effects of price changes on the demand for PICs. The survey was conducted between August and November 2013 in 12 large Indian cities with a population size of more than 500,000 inhabitants (see Fig 2). According to Census data India had 91 cities with a population over 500,000 in 2011, with a total population of 146m [14]. The survey was limited to households with a 6-23-month-old child and all interviews were conducted with the mother of the child. Households where the mother was not responsible for deciding which foods to give to the child were excluded from the survey.

The sample was stratified both by region (north, east, south and west) and city size (cities with a population over 4m, between 1 and 4m and between 0.5 and 1m). This ensures that each region is represented equally in the sample with 1200 respondents. Large and medium sized cities both have an equal sample size of 475 households, while smaller cities have a sample size of 250. The identification of the households within each city followed a systematic random sampling process: Starting households were drawn randomly from the National Electoral Rolls and first contacted as a starting point. Five interviews in households with a 6-23-month-old child were then conducted around each starting point. These households were chosen according to the right hand rule, where every 3^rd^ household was contacted. Even if a household was not eligible for participation, its contacts were maintained. The interviewer continued to contact households according to the right-hand rule until a total of five interviews had been conducted.

The questionnaire was designed by the authors in cooperation with IMRB International (New Delhi, India) and the survey was conducted by IMRB International.

The survey complies with the strict ethical standards of Nestlé India [18], with the IMS Act (1992), and the WHO code on the marketing of breast milk substitutes [19]. The superiority of breast milk on all infant foods was clearly communicated to the respondents, by stating that complementary feeding should not begin before the child reaches 6 months of age, as recommended by the WHO [4]. Households with children below 6 months were not contacted. Breast-feeding status was assessed at the beginning of the interview and mothers who were exclusively breast-feeding were not further interviewed. At no time were any infant foods made available to respondents for free or at reduced prices.

The survey assessed the following: 1) household composition, 2) current general food intake of the child, intake of F-PICs and reasons not to buy F-PICs, 3) price sensitivity of demand which was assessed using a hypothetical buying experiment, 4) nutritional knowledge and attitudes regarding child nutrition, 5) socio-economic characteristics.

The information on socio-economic characteristics was used to determine a wealth index according to DHS methodology [17] and using the same set of variables as in the NFHS-3 [16]. Households were classified into 10 SES, using the same quantile break points as in the DHS data, allowing us to link the information collected in the survey with data from the NFHS-3 [16]. Missing socio-economic information was imputed using non-parametric missing value imputation for mixed-type data as developed by Stekhoven and Bühlmann and implemented in the R-Package missForest [20, 21].

The effect of price subsidies on the demand for F-PICs was assessed with a hypothetical buying experiment. Respondents were first asked to imagine that they were eligible for a program allowing them to buy F-PICs at permanently lower prices until the child turns two. The type of marketing experiment depended on whether the household was currently buying F-PICs or not: *Current buyers* of F-PICs were offered a lower price based on a randomly drawn price discount of 20%, 40%, 60% or 80% on the brand of cereals they usually purchase, and then asked to state the amount of F-PICs they would buy at this reduced price. All others mothers were shown a de-branded 25-gram sachet and told, that according to the program, the 6-23-months-old child should be fed with 2 packages per day (corresponding to 50g per day) until the age of 2 years. They were then asked whether they would buy these sachets at a randomly drawn price of either 14, 11, 8, 5, 2 INR (corresponding roughly to a 0%, 20%, 40%, 60%, and 80% discount on current market price). If the answer was yes, the mothers were asked to state the amount they would buy in a regular week. If the answer was no, they were asked whether they would buy the F-PICs at one of the lower price points. Mothers stating that they would buy at one of these discounted prices were classified as *potential buyers* and the others as *non-buyers*. In order to avoid anchoring effects we estimated price sensitivity based on the information on the first price offered only.

Our buying experiment was purely hypothetical. We thus did not actually sell F-PICs for money in contrary to other studies based on willingness-to-pay experiments for health related products such as bed nets [22] or chlorine [23]. The main reason for running a hypothetical rather than a real experiment was that it is forbidden by Indian law to sell packaged infant foods at discounted prices. Moreover, we wanted to assess the amount of F-PICs that would be bought on a regular basis to estimate how much the child’s average daily consumption of F-PICs would increase. Actually selling discounted F-PICs would most probably have induced a hoarding bias by motivating respondents to buy more F-PICs than they would actually feed to the child in a week. A hypothetical marketing experiment should on the contrary not be affected by this hoarding bias. Hypothetical buying experiments are however affected by acquiescence bias and some respondents are likely to state higher amounts bought than they would have bought in a real world situation. We try to minimize these biases by adopting the certainty scale methodology suggested by Blumenschein, Blomquist [24]: Following the buying experiment mothers were asked how certain they were, that they would actually make the purchase they just declared. If the mother declared not to be certain about her answers we excluded these answers from the subsequent calculations. Such ex-post adjustments have been shown to allow unbiased willingness-to-pay estimates [25].

### Cost-effectiveness estimation of price subsidies

In a third step we calculated the cost-effectiveness of price subsidies on F-PICs in reducing IDA by comparing the net social cost of price subsidies with the DALYs averted with price subsidies. We proceeded as follows; *First*, we estimated the demand effects of price subsidies based on the information from the survey. *Second*, we translated the demand effects of price subsidies into health effects using the results of a systematic review of randomized controlled trials on the effectiveness of F-PICs in reducing anemia [15]. *Third*, we calculated the cost-effectiveness of price subsidies on F-PICs by defining a set of price-based interventions and running our model of the costs of IDA before and after the interventions [9]. We considered interventions differing both by the level of the subsidy and by the share of households eligible, where eligibility is determined by socioeconomic status. The net social cost corresponds to the cost of the subsidy minus the production losses averted by reducing IDA.

We estimated the demand effects of price subsidies by combining the data on current demand at actual prices with the data on hypothetical demand at reduced prices. For each household we have information on price and quantity bought in two different states. Our data thus have a panel data structure which allows us to estimate a fixed effects model. By including individual fixed effects, we eliminated any time invariant heterogeneity, both observed and unobserved. Individual fixed effects control for example for the socioeconomic strata, product knowledge, attitude and any other factor that differs between households, but do not change between the two observed states. Because the only difference between states is the price of the product, we could only estimate the effect of price and of interaction terms with price, which is what we are interested in. We estimated the model separately for current and potential buyers. For current buyers we found that a constant elasticity model provides a better fit than a level model. A similar model cannot be estimated for potential buyers due to the significant number of households buying 0g of F-PIC before and after the intervention. We thus estimated Eq (1) for current buyers and Eq (2) for potential buyers:

$$\text{log}\left(Y_{it}\right)=\beta_{0}\text{log}(P_{it})+\beta_{1}\left({\text{SES}}_{i}\times \text{log}(P_{it}\right))+\theta_{i}+\varepsilon_{it},$$ (1)

$$Y_{it}=\beta_{0}P_{it}+\beta_{1}\left({\text{SES}}_{i}\times P_{it}\right)+\theta_{i}+\varepsilon_{it},$$ (2)

with *Y*_*it*_ representing the weekly amount of F-PIC (in grams) bought by individual *i* at state *t* (before or after the experiment), *P*_*it*_ representing the price for 10g of F-PIC that is paid by individual *i* in state *t*, and SES_*i*_ being an ordinal variable representing the individual’s SES (wealth index decile). Finally, the individual fixed effect *θ*_*i*_ eliminates time invariant unobserved heterogeneity between individuals. Note that the same *β*_0_ and *β*_1_ can be identified if the model is estimated in first differences, i.e. Δ*Y*_*i*_ = *β*_0_Δ*P*_*i*_ + *β*_1_(SES_*i*_ × Δ*P*_*i*_) + Δ*ε*_*i*_. We thus exploit the variation over different states, and not over different individuals to estimate the demand effects. The parameters of interest are *β*_0_ representing the effect of a price change for the lowest SES and *β*_1_ representing the change in the effect of price on quantity as SES increases by 1.

Models including higher orders of price polynomials were tested. However, we did not find a significant improvement in the model. Moreover, specification checks have revealed fairly constant differences between two adjoining SES which is why we include SES as an ordinal variable. More specifically, we compared the estimates of the parametric model as described in Eq (1) with the estimates of a semi-parametric model including 10 dummy variables for SES, and found that the observed variation is captured adequately with our baseline model.

In a next step, the estimated demand effects of price subsidies are translated into health effects using the results found in the systematic review by Eichler et al. [15]. The review finds that the daily consumption of 50g of F-PICs per day (or 350g per week) by 6-23-month-old children increases Hb level by 8.7g/l Hb versus the consumption of non-fortified PICs.

**Fig 3:**
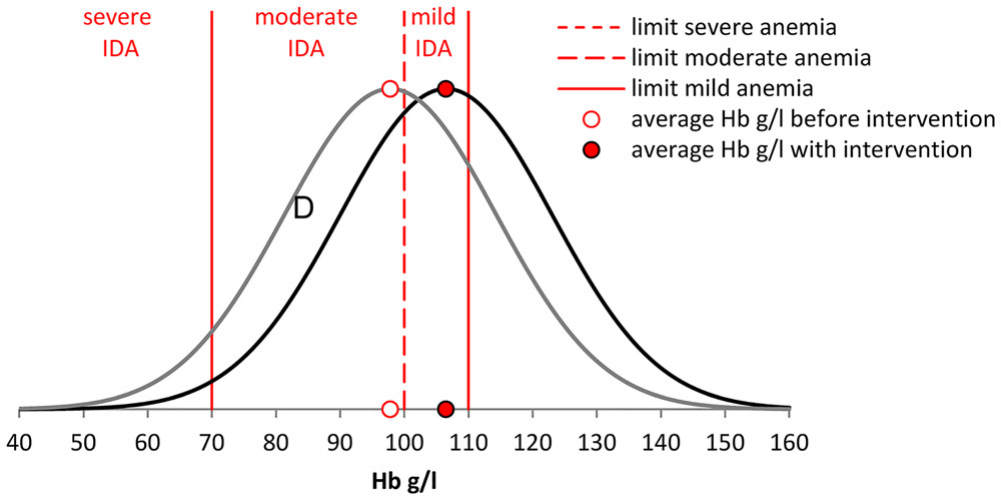
Effect of intervention on the prevalence of IDA. The figure illustrates how the effect of the intervention is modelled for a single SES. We assume the full intervention of 50g per day would move the average hemoglobin level by the 8.7g/l Hb observed in a systematic review on the effectiveness of F-PICs in reducing anemia [15]. The area D corresponds to the reduction of moderate IDA obtained with the intervention. Note that the prevalence of mild anemia would increase with the intervention as the share of children moving from moderate to mild IDA is higher than the share of children moving from mild IDA to no IDA.

Fig 3 illustrates how the effect of the intervention on Hb is modeled for each SES. A full intervention of 50g per day would move the distribution of hemoglobin levels to the right with the average Hb-level increasing by 8.7g/l. As a result, the number of children with severe, moderate, and mild IDA changes. If the change in quantity due to price changes is less than the optimal amount of 50g, we assume that Hb levels increase proportionally.

**Fig 4:**
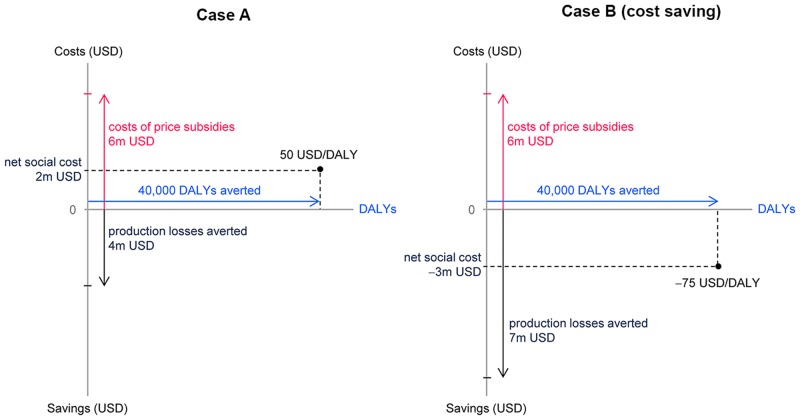
Illustration of cost-effectiveness calculation. The figure illustrates the calculation of cost-effectiveness. Costs are measured in USD and represented on the vertical axis. The upward pointing arrow denotes the cost of the intervention and the downward pointing arrow the production losses averted by the intervention. The net social costs of an intervention are the difference between the costs of the intervention (costs of price subsidy) and the costs averted by the intervention (production losses averted). The DALYs averted by the intervention are represented on the horizontal axis. The cost-effectiveness is assessed by dividing net social costs by the DALYs averted by the intervention.

Finally, we calculated the cost-effectiveness of the price subsidies by first computing the net social costs of an intervention and then dividing these by the amount of DALYs averted. Fig 4 illustrates this procedure with two examples. In case (A) a hypothetical price subsidy of 6m USD reduces production losses by 4m USD and averts 40,000 DALYs. The resulting net social costs amount to 2m USD and the price per DALY averted is 50 USD/DALY (net social costs of 2m / 40,000 DALYs). In case (B) the intervention leads to a stronger reduction in production losses of 7m USD resulting in a negative social cost of −3m USD. The price per DALY averted is negative at −75 USD/DALY. The intervention is therefore cost-saving, as it not only averts DALYs but also saves net social costs. The cost-effectiveness of the intervention would be different if the provider of the subsidy does not take into account the productions losses averted. In this case the cost-effectiveness ratio is calculated by dividing the cost of the price subsidies by the number of DALYs averted, obtaining a cost of 150 USD/DALY for both case (A) and case (B).

## Results

### Social costs of IDA

We calculate the lifetime costs of a birth cohort of children affected by IDA from the age of 6 months to the age of 23 months by adapting the input parameters of Plessow et al. [9] to Indian cities with a population over 0.5m. Table 1 shows the distribution of the birth cohort of 2,798,000 children in 2013 across SES. The number of children per SES decreases with increasing SES, with 14.6% of children living in poorest decile of households and 6.8% in the wealthiest. Table 2 shows mortality rates by wealth quintiles. Mortality rates decrease as household wealth increases, with a 2.8 times higher mortality in the poorest than in the wealthiest quintile.

**Table 1:** Distribution of the birth cohort across SES.

SES deciles (low to high)	1	2	3	4	5	6	7	8	9	10
Share in birth cohort (%)	14.6	11.3	11.0	10.8	10.8	9.5	9.1	8.6	7.4	6.8

Source: Own calculation based on NFHS-3 survey [16] and 2011 Census data [14]

**Table 2:** Mortality rates of 6-23-month-old children across SES.

SES quintiles (low to high)	1+2	3+4	5+6	7+8	9+10	Overall
Yearly mortality per 1000 children	7.0	4.6	3.1	2.4	2.5	4.2

Source: Own calculation based on NFHS-3 survey [16]

**Fig 5:**
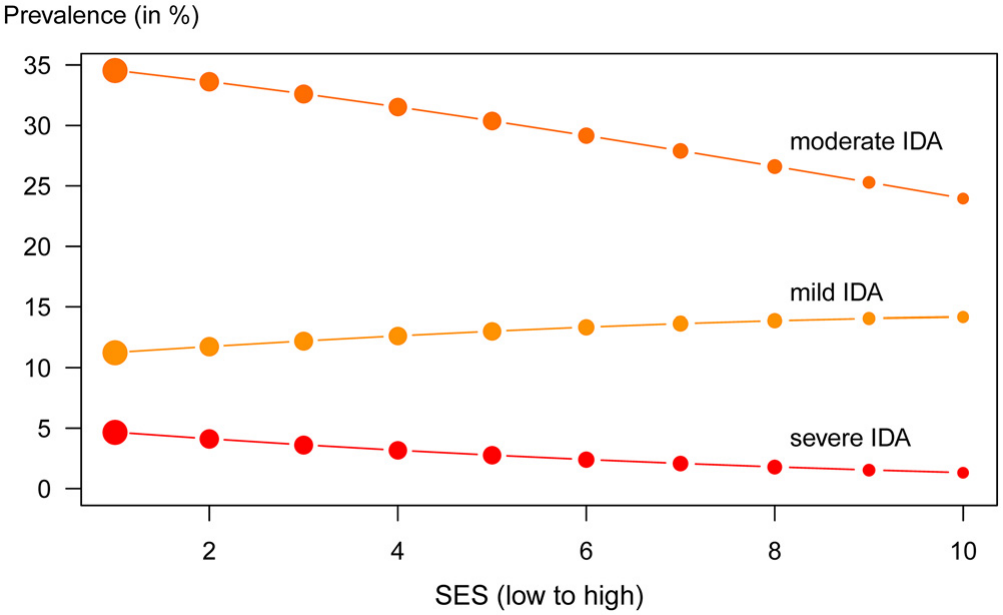
Prevalence of IDA in 6-23-month-old children. The figure shows the share of children suffering from IDA by SES and by degree of severity. Circle size reflects the number of children per SES.

Fig 5 shows the prevalence rates of mild, moderate and severe IDA. The prevalence of moderate and severe IDA decreases across SES, whereas mild IDA is slightly more common in wealthier households. Overall 46% of children are affected by IDA (mild IDA: 12.8%, moderate IDA 30.2%, severe IDA 3.0%).

Table 3 shows the lifetime costs for a birth cohort affected by IDA between the age of 6 and 23 months. Costs are reported by type of costs (production losses and intangible costs) and by time dimension of costs (current costs, future costs, costs due to mortality). Total production losses amount to 3222m USD and are dominated by future losses due to impaired cognitive development. Intangible costs amount to 726,000 DALYs and accrue as current costs during the 6–23 months-age period (13% of total DALYs), as years live lost (6%) as well as future costs in later life (81%).

**Table 3:** Social cost of IDA in large Indian cities due to IDA in 6-23-month-old children.

Production losses (m USD) corresponding to lost future incomes (discounted at 3%)			Intangible Costs (in 1000s) corresponding to health life years lost in terms of DALYs (not discounted)			
Future	Mortality	Total m USD	Current	Future	Mortality	Total DALYs
			(YLD)	(YLD)	(YLL)	
3172	50	**3222**	92	588	46	**726**

YLD: years lived with disability; YLL: years of life lost

**Fig 6:**
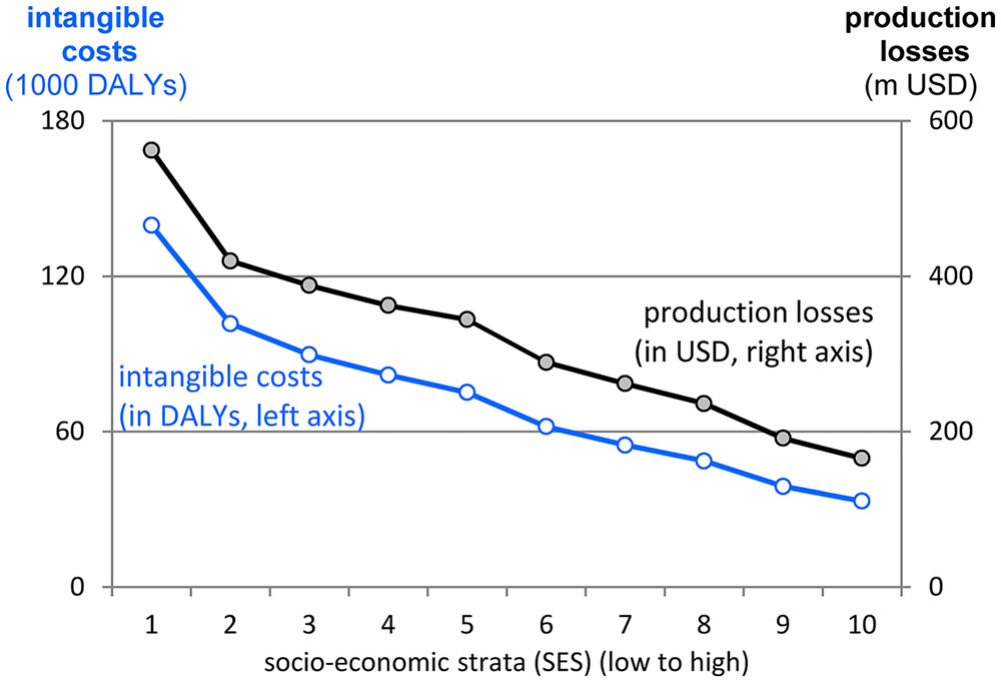
Distribution of social costs across SES. Social costs of IDA are measured as DALYs due to increased morbidity and mortality (intangible costs) and as future incomes lost due to impaired cognitive development (production losses). Both cost categories decrease with increasing wealth due to lower prevalence of IDA and lower number of children per household.

Fig 6 illustrates the distribution of social costs across SES. Both, production losses and intangible costs decrease as wealth increases due to the decreasing number of children per household and the decreasing prevalence of IDA.

### Results of the market survey

In total, 26,630 households were contacted to obtain 4801 complete interviews. In 10% of all attempted contacts no one was at home. The vast majority of unsuccessful contacts (79.8%) were due to the absence of a 6-23-month-old child in the household. Only 0.3% of respondents refused to take part in the interview and only 0.13% of interviews had to be terminated for ethical reasons, because mothers were exclusively breastfeeding. The share of households that could not be contacted or refused participation is at a low 14.0%.

Table 4 shows the number of surveyed households by wealth quintile and city of residence. The number of households varies between 203 in the fifth quintile and 1814 in the third quintile. Households have been classified according to the quantile break points calculated in the DHS data. Hence, reported quintiles are quintiles of the whole population in large cities and not of the population surveyed. Therefore, the distribution of respondents is unbalanced with SES quintiles 2 and 3 overrepresented and the poorest and the wealthiest quintile underrepresented.

**Table 4:** Number of households by SES quintiles.

city size	city	SES 1+2	SES 3+4	SES 5+6	SES 7+8	SES 9+10	Total
	Delhi	34	83	141	169	50	477
over 4 m	Kolkata	67	207	144	46	10	474
	Mumbai	29	129	234	76	9	477
	Chennai	18	113	185	140	12	468
	Lucknow	40	108	166	138	24	476
between 1 and 4 m	Patna	94	188	146	41	5	474
	Pune	48	89	212	119	14	482
	Vizag	42	190	211	27	4	474
	Jalandhar	42	61	72	49	30	254
between 0.5 and 1 m	Bhubaneswar	53	71	104	21	0	249
	Bhilai	32	87	107	13	6	245
	Cochin	1	15	92	104	39	251
	**Total**	**500**	**1341**	**1814**	**943**	**203**	**4801**

Section A in Table 5 reports the characteristics of the selected households across SES. Households differ little in the household size, the number of children under 5, and the age of the referent child. However, the highest level of education achieved by the household head differs substantially, as the share of primary school decreases from 56% in the poorest to 5% in the wealthiest quintile, while the share of college degree increases from 3% to 57%. Furthermore, wealthier households are more often aware of iron in food and are much more likely to have received professional advice on how to feed their child.

**Table 5:** Household characteristics across SES.

	SES 1+2		SES 3+4		SES 5+6		SES 7+8		SES 9+10	
	Mean	SD	Mean	SD	Mean	SD	Mean	SD	Mean	SD
**Panel A:** *Socio-economic characteristics and status of information*:										
Number of household members	4.6	1.7	4.6	1.8	4.6	1.8	4.8	1.8	5.4	2.1
Number of children under 5	1.4	0.6	1.3	0.5	1.2	0.5	1.2	0.5	1.2	0.5
Age of reference child (in months)	14.4	5.17	14.3	5.3	14.6	5.3	14.4	5.3	14.3	5.3
Education household head:										
Primary (in %)	56		47		31		17		5	
High School (in %)	13		32		40		37		37	
College (in %)	3		11		27		45		57	
Fully vegetarian household (in %)	9		9		14		23		33	
Received professional advice	53		69		74		75		72	
Knowledge of iron in food (in %)	57		79		85		93		96	
Monthly household income (USD)^a^	109	67	145	79	225	132	307	169	444	190
**Panel B:** *Current buying and consumption behaviour of packaged infant cereals*:										
**Current buyer:**										
Proportion (in %)	14.0		24.0		31.0		36.0		33.0	
Quantity bought (grams per week)	442	662	478	740	564	823	498	727	552	845
Price paid per 10 gram (in INR)	5.16	1.92	5.10	2.52	5.32	2.35	5.46	2.68	5.69	2.16
**Potential buyer:**										
Proportion (in %)	63		59		50		45		48	
**Non-buyer:**										
Proportion (in %)	23		17		19		20		19	
**Reasons for not buying (in %)** (potential buyers & non-buyers):										
Reason: Monetary	65		51		37		31		18	
Reason: Availability	10		10		14		12		7	
Reason: Advice	2		5		7		9		7	
Reason: Information	19		13		10		6		4	
Nr of observations	500		1341		1814		943		203	

g: gram; SD: standard deviation; SES: socio-economic strata. The recommended daily amount is 50g.

Section B in Table 5 reports information on the current F-PIC purchases. We distinguish between *current buyers*, who bought F-PICs in the last 7 days prior to the interview, *potential buyers*, who would buy F-PICs at any of the prices offered in the hypothetical buying experiment, and *non-buyers*, who would not buy F-PICs even at very low prices (2 INR per 25g pack). The share of *current buyers* is substantially higher among wealthier households with one in three households serving F-PICs in the wealthiest quintile versus one in seven in the poorest. Average quantity bought by current buyers is more or less constant across SES. The average price per 10g increases only slightly across SES as one brand with relatively uniform prices across regions dominates the Indian market for F-PICs. The share of *non-buyers* is almost constant across SES with around 20% of households who would not buy F-PICs even at the lowest price offered. The share of *potential buyers* decreases from 63% in the lowest to 48% in the wealthiest quintile.

Section B in Table 5 also reports the reasons for not buying F-PIC stated by *potential buyers* and *non-buyers*. The main reason is lack of money, which is mentioned by 65% of the poorest and 18% of the wealthiest households. This is a clear indication that a price based intervention might be an effective way to increase the consumption of F-PICs. Lack of information about the product, declared by 19% of the poorest households versus 4% of the wealthiest, may also be a relevant obstacle.

Table 6 shows the results of the panel data estimates for current and potential buyers. The upper section presents coefficient estimates for the model as specified in Eqs 1 and 2. The negative sign for the price effect indicates that quantity bought increases as price decreases. The positive coefficient for the interaction effect indicates that poor households are more price sensitive than wealthier households.

**Table 6:** Estimates of reaction to price change.

Current Buyers		Potential Buyers	
Dependent variable:	log(Quantity bought per week)	Dependent variable:	Quantity bought per week
log(price)	-0.328*** (0.059)	price	−35.000*** (2.018)
log(price) x wealth	0.016 (0.01)	price x wealth	1.557*** (0.379)
*Marginal effects*:		*Marginal effects*:	
mfx_SES1_	-26.44*** (4.19)	mfx_SES1_	-33.44*** (1.67)
mfx_SES2_	-25.12*** (3.41)	mfx_SES2_	-31.88*** (1.35)
mfx_SES3_	-26.33*** (2.96)	mfx_SES3_	-30.32*** (1.06)
mfx_SES4_	-24.88*** (2.27)	mfx_SES4_	-28.77*** (0.84)
mfx_SES5_	-27.31*** (2.11)	mfx_SES5_	-27.21*** (0.77)
mfx_SES6_	-25.62*** (2.09)	mfx_SES6_	-25.65*** (0.87)
mfx_SES7_	-20.82*** (2.25)	mfx_SES7_	-24.09*** (1.10)
mfx_SES8_	-19.35*** (2.94)	mfx_SES8_	-22.54*** (1.39)
mfx_SES9_	-18.84*** (3.96)	mfx_SES9_	-20.98*** (1.72)
mfx_SES10_	-17.29*** (4.88)	mfx_SES10_	-19.42*** (2.07)
Observations	2,354	Observations	4,804
R2	0.127	R2	0.346
Adjusted R2	0.063	Adjusted R2	0.173
F Statistic	85.63*** (df = 2; 1175)	F Statistic	633.480*** (df = 2; 2400)

Note:

*** p < 0.01

The middle section shows marginal effects calculated by combining the general and the SES specific effect of price on quantity. Marginal effects express the change in the quantity of F-PIC bought when the price increases by 1 INR per 10g. We find that both *current* and *potential buyers* increase the amount of F-PIC bought when prices are lowered, with *potential buyers* showing a slightly stronger reaction. Moreover, the results show that poor households are more price sensitive than wealthier ones.

**Fig 7:**
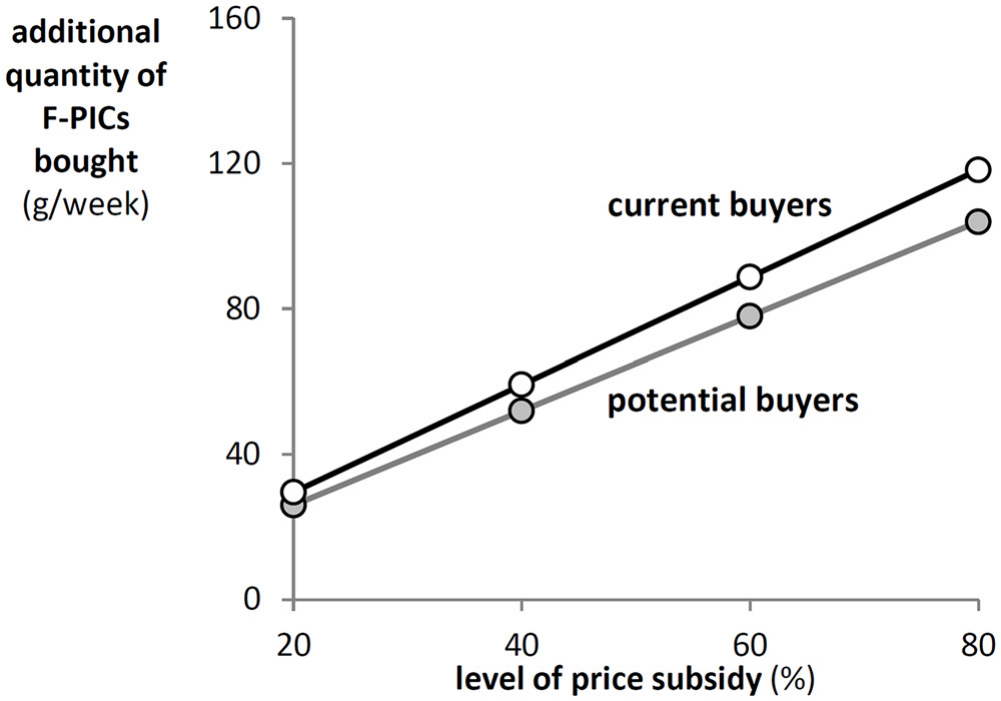
Impact of price subsidies on weekly demand for F-PICs. The figure shows the reaction to price subsidies by current and potential buyers applying the average of the marginal effects and assuming a price of 14 INR per serving of 25g of F-PICs.

Fig 7 shows the average quantity increase at different price subsidies for *current* and *potential buyers* based on the estimated marginal demand effects. Potential buyers react slightly stronger to subsidies. A maximum subsidy of 80% leads to an increase in the quantity bought of 104g per week for *current buyers* (0.6 sachets per day) and of 120g per week for *potential buyers* (0.69 sachets per day). This is an increase in consumption corresponding to approximately a third of the recommended dose of 350g per week.

### Cost-effectiveness of price subsidies

We calculate the costs and the effectiveness of a series of interventions, differing in the share of households eligible for the price subsidy and in the level of the subsidy. The minimal intervention is a price subsidy of 20% for the lowest wealth decile of households. The price subsidies and the share of households eligible for the intervention are then increased stepwise until the whole population is eligible for an 80% price subsidy.

The maximum effect of the subsidy is calculated by increasing the current average hemoglobin (Hb) levels by the amount identified in the systematic review by Eichler et al. [15]. The review finds that the daily consumption of 50g of F-PICs per day (or 350g per week) by 6-23-month-old children increases Hb level by 8.7g/l Hb versus the consumption of non-fortified PICs.

Table 7 reports results of different interventions, varying the level of the price subsidy and the share of households eligible. Section A shows the maximum percentage of DALYs potentially averted with F-PICs, if all children, including those in non-buyer households, would receive the full additional 50g per day. The percentages reported in the columns are cumulative and thus always include all the lower wealth deciles eligible for the subsidy. The last column shows that an intervention covering all SES would prevent 44.6% of the total DALYs due to IDA.

**Table 7:** Percentage DALYs averted by price subsidy as % of total DALYs due to IDA.

	Δ DALY (in %)									
	Highest wealth decile eligible:									
Discount	SES1	SES2	SES3	SES4	SES5	SES6	SES7	SES8	SES9	SES10
***Section A***: *Assuming 100% compliance (no non-buyers)*:										
max	8.3	14.4	19.7	24.7	29.3	33.2	36.7	39.8	42.4	44.6
***Section B***: *Using current and potential buyer share according to survey*:										
20%	0.6	1.2	1.6	2.0	2.4	2.7	2.9	3.1	3.3	3.4
30%	1.0	1.7	2.4	3.0	3.6	4.0	4.3	4.7	4.9	5.1
40%	1.3	2.3	3.2	4.0	4.7	5.3	5.8	6.2	6.5	6.7
50%	1.6	2.9	4.0	5.0	5.9	6.6	7.2	7.7	8.1	8.4
60%	1.9	3.4	4.8	5.9	7.0	7.9	8.5	9.1	9.6	10.0
70%	2.2	4.0	5.6	6.9	8.1	9.1	9.9	10.6	11.2	11.6
80%	2.5	4.5	6.3	7.8	9.2	10.3	11.2	12.0	12.7	13.2
50g/d for free	6.6	11.5	16.1	20.4	24.2	27.5	30.4	33.0	35.2	37.0
***Section C*:** *Share of* ***c****urrent and potential buyers*										
	SES1	SES2	SES3	SES4	SES5	SES6	SES7	SES8	SES9	SES10
Current	0.106	0.155	0.187	0.280	0.307	0.311	0.345	0.380	0.351	0.288
Potential	0.659	0.614	0.630	0.553	0.499	0.505	0.471	0.406	0.477	0.481
Total	0.765	0.769	0.817	0.833	0.806	0.816	0.816	0.786	0.828	0.769

Values in Sections A and B are cumulative, as the percentage of DALYs averted with a highest wealth decile eligible for a price subsidy includes all wealth deciles below this highest wealth decile.

Section B reports the percentage of DALYs averted applying the information collected in the market survey on the share of *current* and *potential buyers* and on the demand response to different price subsidies. The DALYs averted are lower than those in section A for two reasons: *First*, because the 20% of households belonging to the group of *non-buyers* cannot be reached with these interventions. *Second*, because the price subsidy often leads to an increase in the demand for F-PICs below the optimal amount of 50 g/day. A 20% price subsidy given exclusively to the households in SES1 would avert 0.6% of DALYs due to IDA (first line and column in section B). The share of the population eligible for the price discount increases when moving to the right, whereas the size of the price discount increases when moving to the next line. Thus, a price subsidy of 80% for all households would avert 13.2% of DALYs due to IDA.

The last line in section B (50 g/day for free) reports the percentage of DALYs averted if all 6-23-month-old children living in *current* and *potential buyer* households were given 50g of F-PIC for free, and if these 50g/day were consumed *in addition* to the amount of F-PICs currently consumed. This is a rather optimistic assumption, as some of the packages of free F-PICs would probably substitute F-PICs previously purchased on the market. This intervention would avert 37.0% of all DALYs due to IDA.

The costs of price subsidies differ between *potential buyers* and *current buyers* of F-PICs. The price subsidies for *potential buyers* are limited to the quantity of F-PICs purchased as a result of the price subsidy. Conversely, the price subsidies for *current buyers* also subsidize the F-PICs that would have been bought anyway. This is a windfall gain for *current buyers* and simultaneously a useless cost for the provider of the price subsidy, as it does not lead to an increased consumption of F-PICs.

The monetary social costs saved by an intervention correspond to the reduction of the production losses due to IDA. As shown in Table 3 these costs occur predominantly due to impaired cognitive development which leads to lower incomes in adult life.

Table 8 presents the results of these cost-effectiveness calculations for a selection of interventions and provides the single elements of these calculations: Column 1 specifies the SES eligible for the intervention; column 2 specifies the height of the price subsidy; column 3 reports the cost of the intervention; column 4 reports the production losses averted and column 5 the DALYs averted by the intervention. The last two columns show the results of the cost-effectiveness calculations: Column 6 reports the cost-effectiveness ratio from the perspective of a provider of the subsidy, who does not consider the production losses avoid, whereas column 7 reports the cost-effectiveness ratio from a social perspective.

**Table 8:** Results of interventions with different price subsidies and highest SES eligible.

1	2	3	4	5	6	7
SES eligible for intervention	Intervention	Cost of Intervention (m USD)	Production losses averted (m USD)	DALYs averted	Cost of Intervention / DALYs averted	Net social cost / DALYs averted
	20% discount	7.7	21.7	8,462	909	-1,655
SES 1–2	50% discount	27.7	54.6	20,759	1,337	-1,293
	80% discount	58.0	87.7	32,587	1,781	-910
	50g/d for free	127.7	249.4	83,604	1,527	-1,456
	20% discount	19.0	40.4	14,690	1,296	-1,454
SES 1–4	50% discount	63.8	101.3	36,033	1,771	-1,040
	80% discount	128.1	162.7	56,558	2,265	-612
	50g/d for free	249.1	471.1	147,680	1,687	-1,503
	20% discount	34.9	56.6	19,468	1,791	-1,117
SES 1–6	50% discount	110.3	142.0	47,765	2,308	-664
	80% discount	213.4	227.7	74,997	2,846	-191
	50g/d for free	363.2	669.6	199,341	1,822	-1,537
	20% discount	49.4	68.5	22,631	2,183	-844
SES 1–8	50% discount	151.9	171.7	55,554	2,734	-357
	80% discount	288.4	275.1	87,267	3,304	152
	50g/d for free	465.0	836.3	239,314	1,943	-1,551
	20% discount	61.3	77.2	24,778	2,473	-643
SES 1–10	50% discount	185.6	193.3	60,840	3,051	-126
	80% discount	348.9	309.6	95,599	3,649	411
	50g/d for free	550.3	964.6	268,301	2,051	-1,544

Most interventions with price subsidies on F-PICs are cost-saving from a social perspective, as indicated by the negative signs of cost-effectiveness ratios in column 7. Only very large discounts (80%) for large shares of the population (SES 1–8 or more) lead to intervention costs that exceed the production losses averted. All other interventions do thus not only avert DALYs, but the costs of the subsidies are more than compensated by the production losses averted.

The cost-effectiveness ratios relevant from the perspective of a subsidy provider who does not consider the production losses averted in column 6 vary from 843 USD/DALY (20% price subsidy for SES1-2) to 3,341 USD/DALY (80% price subsidy for SES1-8). Whether these interventions are cost-effective or not depends on the cost-effectiveness threshold applied. The WHO recommendations on cost-effectiveness [26] define an intervention as highly cost-effective if the cost per DALY is less than GDP per capita and as cost-effective if the cost per DALY is between one and three times GDP per capita. Applying these thresholds to our results the price subsidies appear highly cost-effective or cost-effective even without considering the production losses averted (Indian per capita GDP was at 1487 USD in 2013 [27]).

Interventions targeted at the poorest deciles of households save most dollars per DALY for three reasons: *First*, IDA is more prevalent and severe in poor households. *Second*, F-PIC demand is more elastic in poor households. An identical price subsidy thus leads to a stronger increase of F-PIC demand in poor households. *Third*, the cost of the price subsidy per child is higher in wealthier SES, as they have a higher share of *current buyers*. Remember, the larger the share of *current buyers*, the more costly the subsidy as it not only subsidizes the additional consumption induced by the price subsidy, but also the current consumption.

The free distribution of 50g/day is cost-saving irrespective of the SES eligible. Note however, that this is a hypothetical scenario which is not directly comparable with the other scenarios. The scenario is based on the assumption that all 6-23-month-old children in *current* and *potential buyer* households consume additional 50g/day of F-PIC. The scenario neglects that not all these additional 50 g/day will lead to an increased consumption of F-PICs. Some *current buyers* may substitute the currently purchased amount of F-PIC with the F-PICs obtained for free without increasing overall consumption. The 50 g/day for free scenario thus represents the maximum effect that may be achieved with full coverage.

## Discussion

### Main results

We estimated the cost-effectiveness of price subsidies on F-PICs in reducing in IDA 6-23-month-old children in large Indian cities by proceeding in three steps: *First*, we assessed the prevalence of IDA and the total lifetime costs in a birth cohort of children affected by IDA between the age of 6 and 23 months; *second*, we carried out a market survey, assessing the current consumption of F-PICs of 4801 households with 6-23-month-old children and their response to price discounts; *third*, we estimated the cost-effectiveness of price subsidies on F-PICs by combining the results of the first two steps with the results of a systematic review on the efficacy of F-PICs in reducing anemia.

Our study adopts a social perspective in evaluating the cost-effectiveness of price subsidies on F-PICs. The social perspective implies that we consider all costs and gains for the society. While the costs accrue to the provider of the subsidy, such as a government agency, the gains accrue to the children covered by the intervention. These gains correspond to the DALYs averted over the whole lifetime of the children and to the production losses averted in their adult working life. Averted production losses correspond to higher incomes and represent a higher overall productivity of the Indian economy due to a healthier population. Our analysis is stratified by 10 SES, which allows us to identify the children most in need.

We find that 46% of 6-23-month-old children are affected by IDA. Severe and moderate IDA are highest in the poorest decile of households (39.2%) but are at striking levels even in the wealthiest quintile (25.3%). The total lifetime costs of IDA in a birth cohort affected by IDA between the age of 6 and 23 months amount to production losses of 3222m USD and intangible costs of 726,000 DALYs in 2013. These DALYs correspond to 10,967 complete lifespans lost or 0.39% of the potential life years of the birth cohort.

The market survey reveals that relatively few households currently buy F-PICs for their child. The share ranges from 14% among the poorest quintile to 36% among richer households. Responses to price subsidies are relatively low. Current buyers increase their weekly consumption by about 25g if prices drop by 1 INR per 10g, while potential buyers only increase consumption by about 30g in response to the same price change.

Additional complementary feeding with F-PICs will not be able to eliminate the entire social costs of IDA. An ideal intervention providing each child with an additional 50g of F-PIC per day would avert 44.6% of DALYs caused to IDA. However, an 80% price subsidy for all households, which is the most comprehensive intervention we modeled, would avert just 13.2% of DALYs. The main reason for this difference is that 20% of *non-buyer* households would not feed their children F-PICs at this discount (*non-buyer* households).

Nearly all interventions with price discounts on F-PICs are cost saving as the production losses averted more than outweigh the cost of the price subsidies. From a social perspective the decision on how much to spend on subsidies on F-PICs should thus be determined by the amount that the provider is able and willing to pay for the subsidy. India is currently spending approximately 1% of its GDP on food programs [28]. With a current GDP of approximately 2,300 billion USD [29] these yearly expenditures on food correspond to 23,000m USD. The cost of an 80% price subsidy on F-PICs covering 60% of households living in large Indian cities amounts to 355.9m USD (see Table 8) and would thus correspond to 1.6% of total cost of food programs in India. Even the most expensive of the interventions we modeled (50g/day for free for 80% of households) would correspond to just 2.4% overall cost of national food programs.

### Results represent lower bound

The results of our cost-effectiveness calculations represent a lower bound of the actual cost-effectiveness of the intervention, because the subsidized F-PICs contain many other micro- and macronutrients besides iron. However, our calculations do not take into account the beneficial effects of these nutrients on child health and physical and cognitive development. Additional calories and proteins are certainly beneficial for many children given the persistently widespread malnutrition in India. In our population, 27.8% of children are malnourished according to height-for-age and 23.2% according to weight-for-age. Additional nutrients are thus highly likely to increase the DALYs and the production losses averted by the intervention. Micronutrients like vitamin A, zinc and iodine may further lead to a decrease in diseases and thus also to a decrease in health care costs. We did not consider these effects in our model, because our knowledge on the prevalence of other nutritional deficiencies in children living in urban India is limited, due to the scarce data available.

### Advantages of market based intervention

The government of India runs a number of vast nutrition programs. The *Public Distribution System* (PDS) provides households in need with subsidized food and fuels through government stores, while the *Integrated Child Development Services* (ICDS) provides nutrition and health care services through Anganwadi centers to preschool children and their mothers. These programs and other welfare initiatives have helped substantially, but they have been unable to solve the problem of malnutrition in India. These programs often struggle to reach those most in need [30] and many of the beneficiaries of the system are not actually poor [31]. These distribution channels are also subject to limitations, such as the Anganwadi centers which according to the 2013 Food Security Act are only allowed to distribute hot cooked food.

Market based interventions have several advantages over interventions distributing products for free. The availability of cheap and wide-spread distribution channels is especially important for F-PICs as they need to be bought on a regular basis. The ease of availability was found to improve uptake [32] and the use existing distribution channels may reduce intervention costs [32]. While a co-payment by the buyer will reduce the uptake of the product it also leads to a selection of buyers who are more likely to actually use the product [23]. Market based interventions can be combined with the targeting to specific groups. Targeting of nutritional products to specific groups is habitual in India [33] and used in many countries for social programs as conditional cash transfers [34].

### Hypothetical nature of the buying experiment

Our study might be criticized for the hypothetical nature of the marketing experiment assessing the price sensitivity of demand for F-PICs, as hypothetical questions are usually subject to bias [35]. However, we could not avoid the use of hypothetical questions for a number of reasons and we designed the questions in order to minimize the potential biases. Willingness-to-pay experiments with F-PICs offered for real money are impossible in India, as the IMS Act bans any advertisements, including free or discounted distribution of food for children under 2 [36]. Furthermore, we are interested in the amount of F-PICs that a household would buy repeatedly, while actual buying experiments always focus on onetime purchases [37, 38]. Our study design also avoids the most important pitfalls of hypothetical questions: F-PICs are readily available for individual purchase and a are well-known product in in large Indian cities. Respondents classified as *potential buyers* were provided with a description of F-PICs and shown a package of F-PIC in order to familiarize with the product. Several of these properties have been shown to improve the validity of purchase intentions, such as the use of existing products, clearly specified products and familiarity with the product [39]. We also apply an ex-post correction for the hypothetical nature of the questions by using a certainty scale that has been shown to effectively adjust hypothetical results to correspond with actual behavior [24, 25].

### Limitations

Our study has a number of limitations: 1) The data on the prevalence of anemia is based on a survey performed in 2005–06. However, there is no indication that the prevalence of anemia in India has decreased in the meantime. It has actually increased between 1998–99 (NFHS-2) and 2005–06 (NFHS-3) and recent studies find comparable prevalence rates [40, 41]. Given the severity and prevalence of malnutrition, there is an urgent need for more frequent data on the health and nutritional status of the Indian population. 2) Our cost-effectiveness calculation doesn’t consider the administrative costs of the intervention. However, these administrative costs are likely to be relatively low if the intervention can be combined with a preexisting intervention targeted at 6-23-month-old children and their mothers. The actual distribution of the F-PICs should lead to no additional costs for the provider of the subsidy as these costs are already included in the current market prices of the product. 3) We provided *potential buyers* with some information on the benefits of F-PIC when assessing their willingness-to-pay for these products. This additional information on the product may have influenced their price sensitivity in addition to the effect of the price discount. However, a real world intervention would most probably be combined with a much stronger emphasis on the importance of nutrient-rich complementary feeding for child health and cognitive and physical development.

## Ethical Statement

This study and the market survey especially adheres to the strict ethical standards of Nestlé India (http://www.nestle.in/brands/mpn/nutrition). It complies with the IMS Act (1992) and the code of marketing of breast milk substitutes by the WHO.

The superiority of breast milk on all infant foods was clearly stated in the communication with respondents. Households with children below 6 months of age were not contacted and it was also clearly stated that complementary feeding should not begin before the child reaches 6 months of age. Breast-feeding status was assessed at the beginning of the interview and respondents who were exclusively breast-feeding were excluded from the survey. At no time were any infant foods made available to respondents for free or at reduced prices.

The market survey has been conducted by IMRB international, a company specialized in market research in India. IMRB handled all the contacts with the participants. The authors did not contact the participants at any time, nor did they have any possibility of identifying respondents during the analysis. Respondents were chosen at random on the condition that they had a 6-23-month-old child and interviewed at home. It was made clear that participation was voluntary and of those willing to participate, informed consent was obtained. The survey focused on patterns of purchasing and consumption of fortified packaged infant cereals. In addition, the survey contained four questions on service use and assessed the nutritional knowledge of mothers.

Ethical Approval was not sought because apart from previously published, completely anonymous and publicly available data, this study is based on market research data only. As opposed to medical research, there is widespread agreement that market research is not subject to ethical approval. For example NHS Health Research Authority states that market research does not require Research Ethics Committee (REC) approval as long as surveys conform to ethical guidelines. EphMRA guidelines also specify that ethical approval is not required for market research. Our study conforms to the ethical standards required by obtaining informed consent and abstaining from any attempts of product promotion. All data is anonymous, any identification of respondents is impossible.

Social cost has been calculated using a previously published model of the social costs of IDA in India [1]. This model is entirely based on publicly accessible data. It combines the information from systematic reviews, journal articles and the world bank database with an analysis of the NFHS-3 dataset. This previously published dataset is completely anonymous and available for public use with no identifiable information on the survey participants. NFHS-3 data is available at http://www.dhsprogram.com/data/available-datasets.cfm”.

Links to Guidelines: **EphMRA:** http://www.ephmra.org/user_uploads/2.%20key%20points%20market%20research%20ethics%20approval_hr.pdf. **NHS HRA:** http://www.hra.nhs.uk/documents/2013/09/does-my-project-require-rec-review.pdf.

## Supporting Information

S1 Questionnaire Recruitment and HH Assessment Questionnaire. (PDF)

S2 Questionnaire Main Questionnaire. (PDF)

## Abbreviations

DALY: disability-adjusted life-year
Fig: Figure
F-PIC: fortified packaged infant cereal
g: gram
g/l: gram per liter
GDP: gross domestic product
Hb: hemoglobin
IDA: iron deficiency anemia
INR: Indian Rupee
m: million
NFHS: National Family Health Survey
SD: standard deviation
SES: socio-economic strata
USD: United States Dollar
YLD: years lived with disability
YLL: years of life lost
