# Supplementary material for: Cost-Effectiveness of Price Subsidies on Fortified Packaged Infant Cereals in Reducing Iron Deficiency Anemia in 6-23-Month-Old-Children in Urban India
Source: PLoS One. 2016 Apr 13;11(4):e0152800. doi: 10.1371/journal.pone.0152800 (PMC4830591; doi:10.1371/journal.pone.0152800)
Supplement: S1 Questionnaire — (PDF) [file pone.0152800.s001.pdf]

PROJECT INFANS - DQ45420
RECRUITMENT AND HOUSEHOLD ASSESSMENT – NEW VERSION

**CENTER**	Delhi	01	Kolkata	02	Mumbai	03	Chennai	04	111-12
	Lucknow	05	Patna	06	Pune	07	Vizag	08	
	Jalandhar	09	Bhubaneshwar	10	Bilai	11	Cochin	12	

**Name of Respondent**		113-132
**Name of the Child (6-23 m)**		133-152
**Address/ Landmark**		
**City**		
**Mobile**		153-162

**Name of Supervisor:**		**Supervisor's Code**				163-164
**Interviewer's Name:**		**Interviewer's Code**				165-166
**Starting Area:**		**SA Code**				187-88

**Interview Number:**				189-91				
**Date of Interview**					**2**	**0**	**1**	**3**	167-170

**ACCOMPANIED**				**BACK CHECKED**				**SCRUTINIZED**			
					P	T					
**TL**	1			**TL**	1	5			**TL**	1	
**EIC**	2			**EIC**	2	6			**EIC**	2	
**OFE**	3			**OFE**	3	7			**OFE**	3	
**FM**	4			**FM**	4	8			**FM**	4	
		171-174					175-182				183-186
**Signature: TL/EIC/OFE/FM**				**Signature: TL/EIC/OFE/FM**				**Signature: TL/EIC/OFE/FM**			

**QUOTA GRID**					
**AGE OF CHILD**		**SEC**		**SEGMENT**	
6-12 months	1	A	1	Current Buyer	1
13-18 months	2	B	2	Non- Buyer	2
19-23 months	3	C	3		
		D	4		
		E	5		
	211		212		213

	LANGUAGE OF QUESTIONNAIRE (SINGLE CODE)	LANGUAGE OF RESPONDENT (SINGLE CODE)	LANGUAGE OF INTERVIEW (SINGLE CODE)
English	01	01	01
Hindi	02	02	02
Bengali	03	03	03
Oriya	04	04	04
Gujarati	05	05	05
Tamil	06	06	06
Kannada	07	07	07
Telugu	08	08	08
Any other (please specify) _________			
	214-215	216-217	218-219

1ST CONTACT DETAILS									
**Date (DD-MM-YYYY):**					2	0	1	3	220-223
**Time of contact (HH-MM):** *PLEASE INDICATE 24 HOUR FORMAT. FOR EXAMPLE, 6:30PM SHOULD BE RECORDED AS 18:30*									224-227
**No one at home**	No one at home		1		Someone at home		2		228
	**CONTACT 2ND TIME**				**CONTINUE**				

APPROACH ANY ADULT & SAY

Good______ or Namaste! I am_______ **(MENTION YOUR NAME)** from IMRB International, a leading market research organization. **Before starting this interview, I wish to confirm that this interview complies with the Market Research Society of India (MRSI) and International code of ethics for market research.** Please be assured that all information given by you will be kept strictly confidential and not revealed to our client with your name/contact details without your prior permission. The response collected will be added together with the responses of others before presenting the findings. Under no circumstance will this information be used for sales or any commercial purpose.

नमस्ते .... मेरा नाम .... है और मै आईएमआरबी इंटरनेशनल, एक अग्रणी मार्किट रिसर्च कंपनी से हूँ। इंटरव्यू शुरू करने से पहले, मै पूष्टि करना चाहूंगा कि यह इंटरव्यू भारत की मार्किट रिसर्च सोसायटी (एमआरएसआई) और मार्किट रिसर्च के लिए नैतिकता के अंतर्राष्ट्रीय कोडस का पालन करके किया जा रहा है। कृप्या आश्वस्त रहे कि आपके द्वारा दी गयी सभी जानकारी पूरी तरह गोपनीय रखी जायेगी और बिना आपकी इजाजत के आपका नाम/ संपर्क विवरण को हमारे किसी क्लाइंट को नही दिया जायेगा। आपसे एकत्र की गयी सभी जानकारी निष्कर्ष पेश करने से पहले अन्य लोगो की प्रतिक्रिया के साथ मिलाई जायेगी। किसी भी परिस्थिति के अंदर यह जानकारी किसी बिक्री या व्यवसायिक उद्देश्य के लिए इस्तेमाल नही की जायेगी।

Do you have any queries before I start the interview? For further clarification, you may also contact my senior at IMRB International at any point during this interview.

मै इंटरव्यू शुरू करूं उससे पहले क्या आपको कोई शंका है? ज्यादा स्पष्टीकरण के लिए आप इंटरव्यू के दौरान किसी भी समय पर आईएमआरबी इंटरनेशनल मे मेरे वरिष्ठ अधिकारी को भी संपर्क कर सकते है।

INTERVIEWER TO CLARIFY AND PROVIDE ASSURANCE

TIME OF START: ___________ **TIME OF END:** __________________ **TOTAL DURATION:** __________

Q101 Is at least one child 6 months to less than 2 years old living in the household? **SINGLE CODING ONLY**
क्या कम से कम एक 6 महीनो से 2 वर्ष से कम उम्र का बच्चा आपके घर मे रहता है?

Yes हाँ	1	**CONTINUE**
No नही	2	**TERMINATE**

229

Q102 Please tell me the name, age and birth date of each mother of children at least 6 months old but less than 2 years old living here. **RECORD DETAILS**
कृप्या मुझे यहां रहने वाले सभी कम से कम 6 महीनो परन्तु 2 वर्ष से कम उम्र के बच्चो की प्रत्येक माँ की जन्मतिथि, नाम और उम्र बताए।

MOTHERS OF CHILDREN IN 6- 23 MONTHS AGE GROUP									
**Sr. No.**	**Name**	**Age (in years)**		**Birth Date (SHOULD BE BETWEEN 1 TO 31)**				**Selected Mother**	
	**RECORD VERBATIM**	**RECORD VERBATIM WITH LEADING ZEROES**						**Circle**	
				**Date**		**Month**			
**1.**								1	230-236
**2.**								2	237-243
**3.**								3	244-250
**4.**								4	251-257
**5.**								5	258-264

IF MORE THAN 1 MOTHER WITH CHILD 6-23 MONTHS OLD LIVING IN THE HOUSE, SELECT THE MOTHER WHO WAS LAST TO HAVE HER BIRTHDAY (MOST RECENT BIRTHDAY)

Q103 Does the mother of the child 6 months to less than 2 years old live here? **SINGLE CODING ONLY**
क्या 6 महीनो से 2 वर्ष की उम्र से कम के बच्चे की माँ यहां रहती है?

Yes हाँ	**1**	**CONTINUE**
No नही	**2**	**TERMINATE**

265

Q104 Is the mother available? May we speak with her? **SINGLE CODING ONLY**
क्या माँ उपलब्ध है? क्या मै उनसे बात कर सकता हूँ?

Yes हाँ	**1**	**CONTINUE**
No नही	**2**	**SECOND CONTACT**

266

IF NOT AVAILABLE, ARRANGE FOR A SUITABLE TIME TO COME BACK.

**2ND CONTACT DETAILS**									
**Date (DD-MM-YYYY):**					2	0	1	3	267-270
**Time of contact (HH-MM):** *PLEASE INDICATE 24 HOUR FORMAT. FOR EXAMPLE, 6:30PM SHOULD BE RECORDED AS 18:30*									271-274
**No one at home**	No one at home	1	Concerned female NOT at home	2	Concerned female at home		3		275
	**TERMINATE**				**CONTINUE**				

TALK TO PROBABILITY RESPONDENT & REPEAT INTRODUCTION

Good______ or Namaste! I am_______ **(MENTION YOUR NAME)** from IMRB International, a leading market research organization. **Before starting this interview, I wish to confirm that this interview complies with the Market Research Society of India (MRSI) and International code of ethics for market research.** Please be assured that all information given by you will be kept strictly confidential and not revealed to our client with your name/contact details without your prior permission. The response collected will be added together with the responses of others before presenting the findings. Under no circumstance will this information be used for sales or any commercial purpose.
नमस्ते .... मेरा नाम .... है और मै आईएमआरबी इंटरनेशनल, एक अग्रणी मार्किट रिसर्च कंपनी से हूँ। इंटरव्यू शुरू करने से पहले, मै पुष्टि करना चाहूंगा कि यह इंटरव्यू भारत की मार्किट रिसर्च सोसायटी (एमआरएसआई) और मार्किट रिसर्च के लिए नैतिकता के अंतर्राष्ट्रीय कोडस का पालन करके किया जा रहा है। कृप्या आश्वस्त रहे कि आपके द्वारा दी गयी सभी जानकारी पूरी तरह गोपनीय रखी जायेगी और बिना आपकी इजाजत के आपका नाम/ संपर्क विवरण को हमारे किसी क्लाइंट को नही दिया जायेगा। आपसे एकत्र की गयी सभी जानकारी निष्कर्ष पेश करने से पहले अन्य लोगो की प्रतिक्रिया के साथ मिलाई जायेगी। किसी भी परिस्थिति के अंदर यह जानकारी किसी बिक्री या व्यवसायिक उद्देश्य के लिए इस्तेमाल नही की जायेगी।

We are carrying out a survey on the food & beverage consumption by children 6 months to less than 2 years of age. The survey will take about 45 minutes.
हम 6 महीनो से 2 वर्ष से कम उम्र के बच्चो द्वारा खाद्य और पेय पदार्थो की खपत पर सर्वे कर रहे है। यह सर्वे करीब 45 मिनट का समय लेगा।

Do you have any queries before I start the interview? For further clarification, you may also contact my senior at IMRB International at any point during this interview.
मै इंटरव्यू शुरू करूं उससे पहले क्या आपको कोई शंका है? ज्यादा स्पष्टीकरण के लिए आप इंटरव्यू के दौरान किसी भी समय पर आईएमआरबी इंटरनेशनल मे मेरे वरिष्ठ अधिकारी को भी संपर्क कर सकते है।

INTERVIEWER TO CLARIFY AND PROVIDE ASSURANCE

Q105

Selected mother is available and agrees to participate चयनित माँ उपलब्ध और हिस्सा लेने के लिए सहमत	**1**	**CONTINUE**	
Selected mother is available but refuses to participate चयनित माँ उपलब्ध परन्तु हिस्सा लेने से मना किया	**2**	**TERMINATE**	**RECORD REASON:**
Selected mother is not available चयनित माँ उपलब्ध नही	**3**	**CONTACT 2ND TIME**	
	**276**		**277-285**

Q106 **SHOWCARD Q106** Thinking about the food/ beverages to buy for your child 6 months to less than 2 years old, which of these statements best applies to you? **SINGLE CODING ONLY**

आपके 6 महीनो से 2 वर्ष से कम उम्र के बच्चे के लिए खाद्‌य/ पेय पदार्थो की खरीदारी के बारे मे सोचते हुए, इनमे से कौन सा वाक्य आपके लिए लागु होता है।

I am the person who **MOST OFTEN** decides and buys for my child what food/ beverage to buy for him/ her	मै वह व्यक्ति हूँ जो बच्चे के लिए क्या खाने/ पिने की खरीदारी और उसके निर्णय मे ज्यादातर शामिल होती हूँ	**1**	**CONTINUE**
I **AM NOT** the person who **MOST OFTEN BUYS** for my child what food/ beverage to buy for him/ her, but I often **ACTIVELY PARTICIPATE** in deciding food/ beverage to buy for him/ her	मै वह व्यक्ति नही हूँ जो ज्यादातर मेरे बच्चे के लिए खाने/ पिने के लिए खरीदारी करता है, परन्तु मै बच्चे के लिए खाने/ पिने की खरीदरी के निर्णय मे सक्रिय रूप से शामिल हूँ	**2**	
I only **SOMETIMES PARTICIPATE** in deciding for my child what food/beverage to buy for him/ her	मै कभी कभी मेरे बच्चो के लिए खाने/ पिने के लिए खरीदरी के लिए निर्णय मे हिस्सा लेती हूँ	**3**	**TERMINATE**
I **NEVER** or **HARDLY EVER** decide for my child what food/ beverage to buy for him/ her	मै मेरे बच्चो के लिए खाने/ पिने के लिए खरीदरी के लिए कम बार या कभी नही निर्णय लेती हूँ	**4**	
		**311**	

SHOW CARD Q107

Q107 Do you or anyone of your family work for any of these companies / organizations? (It could be full time or part time). **MULTIPLE CODING POSSIBLE**

क्या आप या आपके परिवार का कोई सदस्य इनमे से किसी कंपनियों/ संगठनो के लिए कार्य करता है? (यह फूल टाइम या पार्ट टाइम हो सकता है)।

Advertising	विज्ञापन	01	**TERMINATE**
Market research	मार्किट रिसर्च	02	
Marketing	मार्केटिंग	03	
Journalism	पत्रकारिता	04	
Infant food products manufacturer	शिशु खाद्‌य प्रोडक्ट निर्माता	05	
Grocery products Retailer/ Wholesaler	किराना प्रोडक्टस के रिटेलर/ होलसेलर	06	
Infant food products retailer/ wholesaler	शिशु खाद्‌य प्रोडक्टस के रिटेलर/ होलसेलर	07	
Bank	बैंक	08	**CONTINUE**
Railways	रेलवे	09	
Municipality	नगर निगम	10	
None of these	इनमे से कोई नही	99	
		312-317	

Q108 Have you participated in any market research surveys during the **past six months**? **SINGLE CODING ONLY**

पिछले 6 महीनो के दौरान क्या आपने किसी मार्किट रिसर्च सर्वे मे हिस्सा लिया है?

Yes हाँ	1	**TERMINATE**
No नही	2	**CONTINUE**
	318	

HH Composition Assessment

Q206 Please tell me the name, gender, birth date and age of all the children and adults, from youngest to oldest, who usually sleep and eat in your household. I will compile a list starting with the children between 6 and 23 months of age. **RECORD DETAILS.** Please also include any domestic servant who lives in your household.
सबसे छोटे से शुरु करते हुए कृप्या मुझे सभी बच्चो और व्यस्को के नाम, लिंग, जन्मतिथि और उम्र बताए, जो आपके घर मे खाते और सोते है। मै 6 और 23 महीनो की उम्र के बीच बच्चो के साथ शुरु करते हुए सुची को बनाउंगा। कृप्या किसी घरेलु नौकर को भी शामिल करे जो घर मे रहता है।

START WITH CHILDREN 6-23 MONTHS FIRST.
THEN THE RESPONDENT (MOTHER).
THEN ALL OTHER MEMBERS OF THE FAMILY FROM YOUNGEST TO ELDEST.

Q207 **GENDER. SINGLE CODING ONLY**

Q208 **DATE OF BIRTH. RECORD DATE/ MONTH/ YEAR**

RECORD DATE OF BIRTH OF ONLY CHILDREN IN AGE GROUP 6 MONTHS TO 23 MONTHS AND THE SELECTED MOTHER
केवल 6 महीनों से 23 महीनों तक की आयु–समूह में बच्चों और चुनी माँ की जन्मतिथि को दर्ज करें

Q209 **AGE. FOR KIDS LESS THAN 2 YEARS OLD, RECORD AGE IN MONTHS. FOR OTHERS, RECORD AGE IN YEARS**
NOTE TO INTERVIEWER: AGE MUST BE CONSISTENT WITH BIRTH DATE.

Q210 **POSITION IN THE FAMILY. RECORD ACCORDING TO CODES BELOW**

Husband of the respondents/ father of the child बच्चे के पिता/ रिस्पोंडेंट के पति	1
Respondent/ Mother of the child रिस्पोंडेंट/ बच्चे की माँ	2
Child of the respondent/ Son or daughter रिस्पोंडेंट का बच्चा/ बेटा या बेटी	3
Parent of the respondent or her husband/ Grandparent of the child रिस्पोंडेंट या उसके पति के माता पिता/ बच्चे के दादा दादी	4
Other Relatives अन्य रिश्तेदार	5

SELECTED CHILD
IF ONLY 1 CHILD AGED 6-23 MONTHS AND CODED '3' IN Q210, CHOOSE HIM/HER AS SELECTED CHILD

IF MORE THAN 1 CHILD AGED 6-23 MONTHS AND CODED '3' IN Q210: SELECT THE CHILD WHO WAS LAST TO HAVE HIS/HER BIRTHDATE (MOST RECENT BIRTHDATE).

NAME OF SELECTED CHILD: ____

Q210b **SHOWCARD Q210b** Which of the following applies to _______________ **(NAME OF SELECTED CHILD)? SIMGLE CODING ONLY**
...... पर इनमें से कौन सा लागू होते हैं?

I only feed breast milk	मैं केवल स्तनपान कराती हूँ	1	**MAINTAIN RQ AND TERMINATE**
I feed both breast milk and other food/ drink items	मैं स्तनपान और अन्य आहार/पेय सामग्रियों को खिलाती/पिलाती हूँ	2	**CONTINUE**
I feed only other food/ drink items and don't feed breast milk	मैं केवल अन्य आहार/पेय सामग्रियों को खिलाती/पिलाती हूँ और स्तनपान नहीं कराती हूँ	3	

Q211 RELATIONSHIP WITH HEAD OF THE HOUSEHOLD (SOMEONE WHO CONTRIBUTES MOST TO THE HOUSEHOLD INCOME)

Head	मुखिया	01
Wife/ husband	पत्नी/ पति	02
Son/ daughter	बेटा/ बेटी	03
Son in law/ daughter in law	दामाद/ बहु	04
Grand child	पोते पोती	05
Parent	माता पिता	06
Parent in law	सास/ ससुर	07
Brother/ sister	भाई/ बहन	08
Brother in law/ sister in law	देवर/ ननद/ साला/ साली	09
Niece/ nephew	भतीजा/ भतीजी/ भांजा / भांजी	10
Other relative	अन्य रिश्तेदार	11
Adopted/ foster/ step child	गोद लिया/ अनाथ/ सौतेला बच्चा	12
Domestic servant	घरेलु नौकर	13
Other not related	अन्य असंबंधित	14
Don’t know	पता नही	98

	Q206	Q207		Q208			Q209	Q210	Q211		
**Sr. No.**	**Name (RECORD VERBATIM)**	**Male**	**Female**	**Date**	**Month**	**Year**	**Age (RECORD VERBATIM WITH LEADING ZEROES)**	**Position in Family**	**Relationship**	**Selected Child (SINGLE CODE)**	
**1.**		1	2							01	326-341
**2.**		1	2							02	342-357
**3.**		1	2							03	358-373
**4.**		1	2							04	374-389
**5.**		1	2							05	411-426
**6.**		1	2							06	427-442
**7.**		1	2							07	443-458
**8.**		1	2							08	459-474
**9.**		1	2							09	475-490
**10.**		1	2							10	511-526
**11.**		1	2							11	527-542
**12.**		1	2							12	543-558
**13.**		1	2							13	559-574
**14.**		1	2							14	575-590
**15.**		1	2							15	611-626

Q201 Now I would like to ask some questions about you.

What's your name? **RECORD VERBATIM**
आपका नाम क्या है ?

NAME OF RESPONDENT: ______________________

Q202 How old were you at your last birthday? **WRITE EXACT AGE IN THE BOX BELOW AND THEN CODE RELEVANT AGE BAND BELOW. SINGLE CODING ONLY**
आपके पिछले जन्मदिन पर आप कितने वर्ष के थे ?

		319-20

Less than 15 years<br>15 वर्ष से कम	1
15 yrs – 20 yrs	2
21 yrs – 25 yrs	3
26 yrs – 30 yrs	4
31 yrs – 35 yrs	5
36 yrs – 40 yrs	6
41 yrs – 45 yrs	7
46 yrs – 55 yrs	8
Above 55 years<br>55 वर्ष से अधिक	9
	321

Q203 **MARITAL STATUS. SINGLE CODING ONLY**

Currently married<br>वर्तमान विवाहित	1
Married, but gauna not performed<br>विवाहित, परन्तु गौना नही हुआ	2
Widowed<br>विधवा	3
Divorced<br>तलाकशुदा	4
Separated<br>अलग	5
Deserted<br>डेसर्टड	6
Never married<br>विवाहित नही	7
	322

Q204 What is the highest grade you have completed? **SINGLE CODING ONLY**
आपके द्वारा पूरा किया गया शिक्षा का उच्चतम स्तर क्या है ?

Illiterate	01
Illiterate but can read	02
Up to 4 yrs of school	03
5-9 yrs of school	04
SSC / 10TH Class	05
11th Class	06
12th Class / HSC	07
Pursuing College	08
Graduate / Post graduate (Gen)	09
Graduate / Post graduate (professional)	10
	323-24

Q205 Please tell me your working status. **SINGLE CODING ONLY**
कृप्या मुझे आपका कार्य स्तर बताए।

Not working/ homemaker कार्यरत नही/ गृहिणी	1
Working part time/ free lancer पार्ट टाइम कार्यरत/ फ्री लांसर	2
Working full time पूरा समय कार्यरत	3
	325

GO TO THE MAIN INTERVIEW
