# Supplementary material for: Cost-Effectiveness of Price Subsidies on Fortified Packaged Infant Cereals in Reducing Iron Deficiency Anemia in 6-23-Month-Old-Children in Urban India
Source: PLoS One. 2016 Apr 13;11(4):e0152800. doi: 10.1371/journal.pone.0152800 (PMC4830591; doi:10.1371/journal.pone.0152800)
Supplement: S2 Questionnaire — (PDF) [file pone.0152800.s002.pdf]

PROJECT INFANS - DQ45420
Main Questionnaire – NEW VERSION

**CENTER**	Delhi	01	Kolkata	02	Mumbai	03	Chennai	04	711-12
	Lucknow	05	Patna	06	Pune	07	Vizag	08	
	Jalandhar	09	Bhubaneshwar	10	Bhilai	11	Cochin	12	

**Name of Respondent**		
**Name of the Child (6-23 m)**		
**Address/ Landmark**		
**City**		
**Mobile**		

**Name of Supervisor:**				**Supervisor's Code**				713-714
**Interviewer's Name:**				**Interviewer's Code**				715-716
**Starting Area:**				**SA Code**				1888-89
**Interview Number:**				1890-92				

**Date of Interview**					**2**	**0**	**1**	**3**	717-720

**ACCOMPANIED**				**BACK CHECKED**				**SCRUTINIZED**			
					**P**	**T**					
**TL**	1			**TL**	1	5			**TL**	1	
**EIC**	2			**EIC**	2	6			**EIC**	2	
**OFE**	3			**OFE**	3	7			**OFE**	3	
**FM**	4			**FM**	4	8			**FM**	4	
		721-724					725-732				733-736
**Signature: TL/EIC/OFE/FM**				**Signature: TL/EIC/OFE/FM**				**Signature: TL/EIC/OFE/FM**			

**QUOTA GRID**					
**AGE OF CHILD**		**SEC**		**SEGMENT**	
6-12 months	1	A	1	Current Buyer	1
13-18 months	2	B	2	Non Buyer	2
19- 23 months	3	C	3		
		D	4		
		E	5		
	737		738		739

TIME OF START: ___________ **TIME OF END:** __________________ **TOTAL DURATION:** __________

Main Questionnaire

Purchase & Consumption Section

CODE RESPONSES FOR Q301– Q303 IN THE GRID GIVEN AFTER Q303

SHOW PHOTOCARDS

Q301 Please tell me in past 3 days what all has **[REFERENT CHILD]** consumed? Please include all food items and beverages the child has consumed. **MULTIPLE CODING POSSIBLE**
कृप्या मुझे बताए पिछले 3 दिनो मे .... ने क्या सब खाया था? कृप्या सभी खाद्य वस्तुओं और पेय पदार्थो को शामिल करे जो कि बच्चे ने खायी/ पी है।

Please do not include today's day when we say past 3 days. Please include 3 days with third day ending yesterday night – 12am.
कृप्या आज का दिन न मिलाएं। पिछले 3 दिनो के बारे मे बताए जो कल रात 12 बजे खतम हुए हों।

SHOW PHOTOCARD FOR EACH CATEGORY CODED IN Q301

Q303 **SHOWCARD Q303** Please tell me approx. how many servings of each item does the child eat in a week? **SINGLE CODING ONLY**
कृप्या मुझे बताए अंदाजन एक सप्ताह मे बच्चा प्रत्येक वस्तु की कितनी सर्विंग खाता है?

SHOW CARD Q303		
6 times per day or more	प्रति दिन 6 या ज्यादा बार	01
5 times per day	प्रति दिन 5 बार	02
4 times per day	प्रति दिन 4 बार	03
3 times per day	प्रति दिन 3 बार	04
2 times per day	प्रति दिन 2 बार	05
1 time per day	प्रति दिन 1 बार	06
5-6 times per week	प्रति सप्ताह 5–6 बार	07
3-4 times per week	प्रति सप्ताह 3–4 बार	08
1-2 times per week	प्रति सप्ताह 1–2 बार	09
Less often	कम बार	10
During emergencies/ special occasions	आपातस्थितियों/ विशेष अवसरो के दौरान	11
Don't know / remember	पता नही/ याद नही	98

Q301			Q303		
**Name of Item Consumed in Past 3 Days**		**Item Code**	**Servings**		
**Breast Milk**	माँ का दूध	084			740-45
Cereals like **Daliya/ Khichdi/ Mixed Grains/ Sewiya**	सीरियल्स जैसे दलिया/खिचड़ी/मिले अनाज/सेवईंया	151			746-51
Cereals like **Ragi/ Barley/ Bajra/ Maize/ Corn**	सीरियल्स जैसे रागी/जौं/बाजरा/मक्का/दाना	152			752-57
**Oats/ Corn flakes/ Rice flakes**	ओट्स/ कोर्नफ्लेक्स/ चावल के फ्लेक्स	153			758-63
**Rice/ Rice based dishes**	चावल/ चावल से बने व्यंजन	154			764-69
**Packaged Infant Cereals**	पैकेज्ड इनफेंट सीरियल्स/ बेबी फूड	155			770-75
**Legumes** like pulses, chana etc.	फली जैसे दालें, चना आदि	156			776-81
**Roti, Paratha, Bread, Dosa, Poori etc.**	रोटी, पराठा, ब्रेड, डोसा, पूरी आदि	157			782-87
**Packaged Powdered Milk**	पैकेज्ड पाउडर्ड दूध	085			811-16
**Homemade Snacks** like poha, semolina/ suji/ rawa, halwa, upma, idli, puffed rice, Idiyappam, sattu, chattuah, sabudana etc.	घर में बने स्नेक्स जैसे पोहा, सेमोलिना/सूजी/रवा, हलवा, उपमा, इडली, फूले चावल, इडियापन, सत्तू, छतूआ, साबुदाना आदि	158			817-22
**Cow milk/ buffalo milk (packaged or loose)**	गाय का दूध/भैंस का दूध (पैकेज्ड या खुला)	159			823-28
**Milk products** like curd, raita, lassi/ chaach, cream, tea, coffee, paneer, khoya, shakes, ice cream etc	दूध के प्रोडक्ट्स जैसे दही, रायता, लस्सी/छाछ, क्रीम, चाय, कॉफी, पनीर, खोवा, शेक्स, आईसक्रीम आदि.	160			829-34
**Non vegetarian foods and non veg soups**	माँसाहारी खाने और माँसाहारी सूप्स	161			835-40
**Infant formulas**	इनफेंट फार्मूला	162			841-46
**Fruits/ Fruit Juices/ Neembu pani**	फल/ फलों के जूस/ नींबू पानी	163			847-52
**Packaged snacks/ foods** like biscuits, chips, soft drinks, confectionary items, namkeens, milk additives likes Horlicks etc.	पैकेज्ड स्नेक्स/फूड्स जैसे बिस्कुट्स, चिप्स, सॉफ्ट ड्रिंक्स, कॉन्फेक्शनरी सामग्रियाँ, नमकींस, दूध एडीटिव्स जैसे हॉरलिक्स आदि	164			853-58
**Vegetables and vegetable soups**	सब्जियाँ और सब्जियों के सूप्स	165			859-64
Others (**PLEASE SPECIFY**) अन्य (कृप्या स्पष्ट करें) __________					1077-82
Others (**PLEASE SPECIFY**) अन्य (कृप्या स्पष्ट करें) __________					1083-88
Others (**PLEASE SPECIFY**) अन्य (कृप्या स्पष्ट करें) __________					1111-16
Others (**PLEASE SPECIFY**) अन्य (कृप्या स्पष्ट करें) __________					1117-22
Others (**PLEASE SPECIFY**) अन्य (कृप्या स्पष्ट करें) __________					1123-28
Others (**PLEASE SPECIFY**) अन्य (कृप्या स्पष्ट करें) __________					1129-34
Others (**PLEASE SPECIFY**) अन्य (कृप्या स्पष्ट करें) __________					1135-40
Others (**PLEASE SPECIFY**) अन्य (कृप्या स्पष्ट करें) __________					1141-46
Others (**PLEASE SPECIFY**) अन्य (कृप्या स्पष्ट करें) __________					1147-52
Others (**PLEASE SPECIFY**) अन्य (कृप्या स्पष्ट करें) __________					1153-58

**CLASSIFY AS:**				
**If [REF CHILD] has consumed packaged infant cereal in last 3 days**	Coded 155 in Q301	**CURRENT BUYER**	1	**GO TO Q304**
**Else**	NOT coded 155 in Q301	**NON BUYER**	2	**GO TO Q316**

1159

ASK Q304-Q315 IF PACKAGED INFANT CEREAL (155) CODED IN Q301

CODE RESPONSES FOR Q304– Q307 IN THE GRID GIVEN AFTER Q307

Q304 What was the brand of packaged infant cereal/ baby foods that was given to **[REFERENT CHILD]? RECORD VERBATIM**
.... को दिए गए पैकेज्ड शिशु अनाज/ शिशु आहार का ब्रांड क्या था ?

Q305 **SHOWCARD Q305** Please look at this card and tell me in which pack type did you buy _______________ **(MENTION BRANDS CODED IN Q304 ONE BY ONE)? SINGLE CODING ONLY FOR EACH BRAND**
कृप्या इस कार्ड को देखे और मुझे बताए आपने .... को किस पैक प्रकार मे खरीदा था ?

**SHOW CARD Q305**		
Poly-pack	पॉली पैक	01
Can	कैन	02
Tin	टिन	03
Plastic pack	प्लास्टिक पैक	04
Tetra pack	टैट्रा पैक	05
Paper pack	पेपर पैक	06
Sachet	सैशे	07
PET bottle	पैट बोतल	08
Glass bottle	ग्लास की बोतल	09
Bagging Box	बैगिंग बॉक्स	10

Q306 And what size did you buy of _______________ **(MENTION BRANDS CODED IN Q304 ONE BY ONE)**?
और आपने .... का क्या साइज खरीदा ?

PLEASE ASK RESPONDENT TO SHOW THE PACK IF AVAILABLE AT HOME AND NOTE PACK SIZE FROM THE PACK.

IF PACK NOT AVAILABLE OR RESPONDENT REFUSED TO SHOW THE PACK, RECORD

PLEASE RECORD THE PACK SIZE IN GRAMS. RECORD VERBATIM WITH LEADING ZEROES

Q307 a) Now please tell me out of ____________ **(MENTION BRANDS CODED IN Q304 ONE BY ONE)** which cereal/ baby food does **[REFERENT CHILD]** consume most? **SINGLE CODING ONLY**
अब कृप्या मुझे बताए .... मे से कौन सा अनाज/ शिशु आहार .... ज्यादातर खाता/पीता है ?

**Q304**	**Q305**		**Q306**						**Q307a**	
			**Pack Size (ml or gram)**							
**Brand name**	**Pack Type**		**RECORD PACK SIZE IN GRAMS (IF CAN NOT BE ASCERTAINED RECORD 9898)**				**NOT AVAILABLE AT HOME CURRENTLY**	**REFUSED TO SHOW**	**MOST OFTEN CONSUMED PACKAGED INFANT CEREAL**	
							1	2	1	1161-71
							1	2	2	1172-82
							1	2	3	1211-21
							1	2	4	1222-32
							1	2	5	1233-43

Q307 **b)** When was the last time when you served packaged infant cereals to_____________ **(REFERENT CHILD)? SINGLE CODING ONLY**
पिछली बार कब आपने .... के लिए पैकेज्ड शिशु अनाज को सर्व किया था ?

Same day	उसी दिन	1
Yesterday	कल	2
2 days back	2 दिन पहले	3
3 days back	3 दिन पहले	4

1244

INSTRUCTION TO INTERVIEWER: PLEASE ASK RESPONDENT TO BRING THE SPOON THEY NORMALLY USE TO POUR PACKAGED INFANT CEREAL WHILE PREPARING FOR CHILD

ALSO, TAKE OUT THE SEMOLINA PACKET

Q308 I have with me some semolina. Please tell me with the help of this spoon, how much quantity of packaged infant cereal you gave to_____________ **(REFERENT CHILD)** during this occasion?
मेरे पास कूछ सेमोलिना/ सूजी है। कृप्या मुझे इस चम्मच की मदद से बताए, पैकेज्ड शिशु अनाज की कितनी मात्रा आप ने इस अवसर के दौरान .... को दी ?

ASK THE RESPONDENT TO PUT THE SEMOLINA IN SPOON. MEASURE THE QUANTITY USING STANDARD BEAKER AND RECORD BELOW

QUANTITY LEVEL			

1246-48

SAY: Now, I'll ask you some questions about the packaged infant cereal/ baby food that is consumed by the child most often i.e. _____________ **(MENTION CEREAL NAME CODED IN Q307)**
कहेः अब, मै आपसे पैकेज्ड शिशु अनाज/ शिशु आहार के बारे मे कूछ प्रश्न पूछूंगा जो कि बच्चे द्वारा ज्यादातर खाया जाता है मतलब ....।

Q309 **SHOWCARD Q309** Please tell me approx. how many servings does the child eat of this packaged infant cereal/ baby food in a week? **SINGLE CODING ONLY**
कृप्या मुझे बताए अंदाजन एक सप्ताह मे इस पैकेज्ड शिशु अनाज/ शिशु आहार की कितनी सर्विंग बच्चा खाता है ?

6 times per day or more	प्रति दिन 6 या ज्यादा बार	01
5 times per day	प्रति दिन 5 बार	02
4 times per day	प्रति दिन 4 बार	03
3 times per day	प्रति दिन 3 बार	04
2 times per day	प्रति दिन 2 बार	05
1 time per day	प्रति दिन 1 बार	06
5-6 times per week	प्रति सप्ताह 5–6 बार	07
3-4 times per week	प्रति सप्ताह 3–4 बार	08
1-2 times per week	प्रति सप्ताह 1–2 बार	09
Less often	कम बार	10
During emergencies/ special occasions	आपातस्थितियों/ विशेष अवसरो के दौरान	11

1249-50

INSTRUCTION TO INTERVIEWER: NOW AGAIN TAKE THE SPOON THE RESPONDENT NORMALLY USES TO POUR PACKAGED INFANT CEREAL WHILE PREPARING FOR CHILD

ALSO, TAKE OUT THE SEMOLINA PACKET

Q310 Now please tell me with the help of this spoon, how much quantity of packaged infant cereal you generally use while making one serving for the child.
अब कृप्या इस चम्मच की मदद से मुझे बताए, पैकेज्ड शिशु अनाज की कितनी मात्रा आप सामान्यता बच्चे के लिए एक सर्विंग बनाते समय उपयोग करते है।

ASK THE RESPONDENT TO PUT THE SEMOLINA IN SPOON. MEASURE THE QUANTITY USING STANDARD BEAKER AND RECORD BELOW

QUANTITY LEVEL			

1251-53

Q311 **SHOWCARD Q311** Please look at this card and tell me which of the following applies to the way this packaged infant cereal/ baby food is generally prepared? **SINGLE CODING ONLY**
कृप्या इस कार्ड को देखे और मुझे बताए निम्नलिखित मे से कौन सा इस पैकेज्ड शिशु अनाज/ शिशु आहार को बनाने के तरीके पर लागु होता है ?

Mixed with normal water	सामान्य पानी के साथ मिलाकर	01
Mixed with warm water	गुनगुने पानी के साथ मिलाकर	02
Mixed with hot water	गर्म पानी के साथ मिलाकर	03
Mixed with normal milk	सामान्य दूध के साथ मिलाकर	04
Mixed with warm milk	गुनगुने दूध के साथ मिलाकर	05
Mixed with hot milk	गर्म दूध के साथ मिलाकर	06
Any other (please specify) __________	कोई अन्य (कृप्या स्पष्ट करे)	

1254-55

Q312 Please look at this card and tell me, **SHOWCARD Q312** how long back did you buy this packaged infant cereal/ baby food? **SINGLE CODING ONLY**
कृप्या इस कार्ड को देखे और मुझे बताए, कितने समय पहले आपने इस पैकेज्ड शिशु अनाज/ शिशु आहार को खरीदा था ?

In last 1 week	पिछले 1 सप्ताह मे	1
In last 2 weeks but not in last 1 week	पिछले 2 सप्ताह मे परन्तु पिछले 1 सप्ताह मे नही	2
In last 1 month but not in last 2 weeks	पिछले 1 महीने मे परन्तु पिछले 2 सप्ताह मे नही	3
In last 2 months but not in last 1 month	पिछले 2 महीनो मे परन्तु पिछले 1 महीनो मे नही	4
In last 6 months but not in last 2 months	पिछले 6 महीनो मे परन्तु पिछले 2 महीनो मे नही	5
Before last 6 months	पिछले 6 महीनो से पहले	6

1256

Q329 Please look at this card and tell me, **SHOWCARD Q329**, from which place do you purchase this packaged infant cereal/ baby food **most often**? **MULTIPLE CODING POSSIBLE**
कृपया इस कार्ड को देखें औ ार मुझे बताये कि आप किन जगहों से इस पैकेज्ड इनफेंट सीरियल/बेबी फूड को ज्यादातर खरीदती हैं ?

		Q329
Kirana store near the locality	क्षेत्र के निकट किराना स्टोर	01
Grocery store/ General merchant	ग्रोसरी स्टोर/ जनरल मर्चेंट	02
Modern format outlet	आधुनिक फॉर्मेट आउटलेट	03
Pharmacy/ Chemist shop	फार्मसी/ कैमिस्ट शॉप	04
From doctor	डॉक्टर से	05
Street vendors	स्ट्रीट वैंडर्स	06
Dairy/ Milk Outlet	डेयरी/ दूध की दूकान	07
Bakery	बेकरी	09
Any other (please specify) ____________	कोई अन्य (कृप्या स्पष्ट करे)	
Any other (please specify) ____________	कोई अन्य (कृप्या स्पष्ट करे)	

1472-73

Q313 **SHOWCARD Q313** Please tell me why do you serve packaged infant cereal/ baby food to **[REFERENT CHILD]**? **MULTIPLE CODING POSSIBLE**
कृपया मुझे बताए आप .... के लिए पैकेज्ड शिशु अनाज/ शिशु आहार क्यों सर्व करते है ?

They are healthy for child	यह बच्चो के लिए स्वास्थ्यदायक है	01
The doctor/ health professional recommended this	डॉक्टर/ स्वास्थ्य पेशेवर ने इसकी सिफारिश की थी	02
My child likes the taste of packaged infant cereal/ baby foods	मेरा बच्चा पैकेज्ड शिशु अनाज/ शिशु आहार के स्वाद को पसंद करता है	03
My child gets digestive problem after eating other foods	अन्य खाने के बाद मेरे बच्चे को पाचन की परेशानी हो जाती है	04
My child is too young for other foods	मेरा बच्चा अन्य आहार के लिए बहुत छोटा है	05
My child is too old for only milk	केवल दूध के लिए मेरा बच्चा बहुत बडा है	06
I don't prefer homemade cereals	मै होममेड सीरियल को पसंद नही करती	07
I don't have time to prepare homemade cereals	मेरे पास बच्चे के लिए घर पर बना अनाज बनाने का समय नही है	08
Any other (please specify) ____________	कोई अन्य (कृप्या स्पष्ट करे)	
Any other (please specify) ____________	कोई अन्य (कृप्या स्पष्ट करे)	

1257-72

Q314 Please look at this card and tell me, **SHOWCARD Q314** who else in your household consumes the packaged infant cereal/ baby food that is prepared for the kids 6-23 months old. **MULTIPLE CODING POSSIBLE**
कृप्या इस कार्ड को देखे और मुझे बताए, आपके परिवार मे और कौन पैकेज्ड शिशु अनाज/ शिशु आहार को खाता/ पीता है जो कि 6–23 महीने की उम्र के लिए बनाया जाता है।

Kids between 2-5 years	2–5 वर्ष के बीच के बच्चे	1
Kids between 6-12 years	6–12 वर्ष के बीच के बच्चे	2
Kids between 13-17 years	13–17 वर्ष के बीच के बच्चे	3
Adults between 18-25 years	18–25 वर्ष के बीच के व्यस्क	4
Adults between 26-40 years	26–40 वर्ष के बीच के व्यस्क	5
Adults above 40 years	40 वर्ष से बडे के व्यस्क	6
No one else consumes packaged infant cereal/ baby food prepared for kids 6-23 months old	6–23 महीनो के बच्चो के लिए तैयार पैकेज्ड शिशु अनाज/ शिशु आहार को कोई और नही खाता पीता	9

1273-78

Q315 Thinking about the total packaged infant cereal/ baby food consumed in your household in last 1 week, please divide it among members in your household who consume packaged infant cereal/ baby food as per their share in total quantity consumed. **RECORD VERBATIM FOR EACH MEMBER CODED IN Q314. RECORD VERBATIM FOR THE SELECTED CHILD. RECORD WITH LEADING ZEROES**
पिछले 1 सप्ताह मे आपके परिवार मे खाए गए कूल पैकेज्ड शिशु अनाज/ शिशु आहार के बारे मे सोचते हुए, कृप्या इस आपके परिवार मे सदस्यों के बीच जो पैकेज्ड शिशु अनाज/ शिशु आहार को खाते है खपत की गयी कूल मात्रा मे उनके हिस्से के अनुसार बांटे।

		Post code from Q314	% Share		
Kids between 2-5 years	2–5 वर्ष के बीच के बच्चे	1			1279-81
Kids between 6-12 years	6–12 वर्ष के बीच के बच्चे	2			1282-84
Kids between 13-17 years	13–17 वर्ष के बीच के बच्चे	3			1285-87
Adults between 18-25 years	18–25 वर्ष के बीच के व्यस्क	4			1288-90
Adults between 26-40 years	26–40 वर्ष के बीच के व्यस्क	5			1311-13
Adults above 40 years	40 वर्ष से बडे के व्यस्क	6			1314-16
Kids between 6-23 months	6–23 महीनो के बच्चे	7			1317-19
			100%		
➔ GO TO Q324					

ASK Q316-Q320 IF PACKAGED INFANT CEREAL (155) NOT CODED IN Q301 (PACKAGED INFANT CEREAL NOT SERVED IN PAST 3 DAYS)

CODE RESPONSES FOR Q316– Q317 IN THE GRID GIVEN AFTER Q317

Q316 **DO NOT EXPOSE THE LIST** Please tell me which brands of packaged infant cereals/ baby foods are you aware of? **MULTIPLE CODING POSSIBLE**
कृप्या मुझे बताए पैकेज्ड शिशु अनाज/ शिशु आहार के किन ब्रांडस से आप अवगत है?

Q317 At what price is a pack of packaged infant cereal/ baby food is available in market? Please specify the pack size as well (in gm). **RECORD VERBATIM WITH LEADING ZEROES**
पैकेज्ड शिशु अनाज/ शिशु आहार का पैक बाजार मे किस कीमत पर उपलब्ध है? कृप्या पैक साइज को भी स्पष्ट करे।

Q316			Q317.1	Q317.2
DO NOT EXPOSE THE LIST/ DO NOT PROMPT			PRICE (in Rs.)	PACK SIZE (in gm)
Cerelac	सेरेलक	01		
Nestum	नेस्टम	02		
Farex	फैरेक्स	03		
Gerber	जरबर	04		
Any other __________	कोई अन्य			
Any other __________	कोई अन्य			
Any other __________	कोई अन्य			
Not aware/ Cannot recall	अवगत नही/ याद नही	98		
		1320-27	1328-31	1332-35

Q318 Have you ever served packaged infant cereals/ baby foods to **[REFERENT CHILD]**? **SINGLE CODING ONLY**
क्या आपने कभी .... को पैकेज्ड शिशु अनाज/ शिशु आहार को सर्व किया है?

Yes<br>हाँ	1	➔ GO TO Q321
No<br>नही	2	➔ GO TO Q319

1336

ASK Q319/ Q320 IF '2' CODED IN Q318

Q319 **SHOWCARD Q319** You said that you never served packaged infant cereals/ baby foods to **[REFERENT CHILD]**. May I know the reason why not? **MULTIPLE CODING POSSIBLE**
आपने बताया कि आपने कभी ………………… को लिए पैकेज्ड शिशु अनाज/ शिशु आहार को सर्व नही किया। क्या मै कारण जान सकता हूँ?

		Q319
I cannot afford packaged infant cereal/ baby food	मै पैकेज्ड शिशु अनाज/ शिशु आहार का वहन/ अफोर्ड नही कर सकता है	01
It is too expensive as compared to homemade food	यह घर पर बने आहार की तुलना मे बहुत महंगा है	02
There is no small pack to try it and if my child does not eat it, it will be a waste of money	इसे आजमाने के लिए छोटा पैक नही है और यदि मेरा बच्चा इसे नही खाता, यह पैसे की बर्बादी होगी	03
It's not healthy for my child	यह मेरे बच्चे के लिए स्वास्थ्यदायक नही है	04
My child doesn't like it	मेरा बच्चा इसे पसंद नहीं करता है	06
My child is too young for it	मेरे बच्चा इसके लिए बहुत छोटा है	07
My child is too old for it	मेरा बच्चा इसके लिए बहुत बडा है	08
My child gets digestive problem after eating it	इसे खाने के बाद मेरे बच्चे को पाचक परेशानी हो गयी	09
Homemade food has much more variety and child requires variety	घर पर बने आहार मे ज्यादा वैरायटी होती है और बच्चे को वैरायटी चाहिए	12
It does not provide fat	यह फैट प्रदान नही करता है	13
My mother-in-law/ other family members/ friends are against it	मेरी सास/अन्य परिवार के सदस्य/दोस्त इसके विरोध में हैं	26
The doctor advised me not to feed packaged food to my child	डॉक्टर ने मेरे बच्चे को पैकेज्ड आहार नही खिलाने की सलाह दी थी	16
Do not have knowledge about all benefits it provides	इसके द्वारा दिये जाने वाले सभी फायदों के बारे में नहीं जानती	17
It cannot be prepared and kept for a few hours like home food, it has to be eaten immediately	इसे घर पर बने आहार की तरह बनाकर कुछ घंटो के लिए नही रखा जा सकता, इसे तुरंत खाना होता है	18
It is raw/ it is not made by boiling or cooking the food	यह कच्चा है/ यह खाना को पकाने या उबालने द्वारा नही बनाया जाता	19
It may contain preservatives which are harmful	इसमे प्रतिरक्षक/प्रिजर्वेटिव हो सकते है जो हानिकारक है	20
The child will not develop habit of eating homemade food for later in life	जीवन मे बाद के लिए बच्चे मे घर पर बने आहार को खाने की आदत विकसित नही होगी	22
It is very sticky and sticks into the mouth of the child	यह बहुत चिपचिपा है और बच्चे के मुहं मे चिपकता है	24
I feel guilty when I give packaged food to my child	मेरे बच्चे को पैकेज्ड आहार देते समय मै दोषी महसूस करती हूँ	25
Not available in the places where I go for shopping	उस जगह पर उपलब्ध नही जहां से मै खरीदारी करने के लिए जाती हूँ	05
Any other (please specify) ______________	कोई अन्य (कृप्या स्पष्ट करे)	
Any other (please specify) ______________	कोई अन्य (कृप्या स्पष्ट करे)	

1337-58

Q320 Would you buy packaged infant cereals/ baby foods if you could afford it? **SINGLE CODING ONLY**
क्या आप पैकेज्ड शिशु अनाज/ शिशु आहार को खरीदेंगें यदि आप इसका वहन/ अफोर्ड कर सके?

Yes हाँ	01	➔ GO TO Q324
No नही	02	
Don't know **(DO NOT READ)** पता नही	98	

1359-60

ASK Q321 IF '1' CODED IN Q318

Q321 Have you <u>ever bought</u> packaged infant cereals/ baby foods for **[REFERENT CHILD]**? **SINGLE CODING ONLY**
Assign 10 class-points if YES (circle number):
क्या आपने कभी ………………. के लिए पैकेज्ड शिशु अनाज/ शिशु आहार को खरीदा है?

Yes हाँ	01	➔ GO TO Q322
No नही	02	➔ GO TO Q323
Don't know **(DO NOT READ)** पता	98	➔ GO TO Q322

नही		

1361-62

ASK Q322 IF PACKAGED INFANT CEREAL (155) NOT CODED IN Q301 AND '1'/'98' CODED IN Q321

Q322 **SHOWCARD Q322** Could you please tell me why you do not give packaged infant cereals/ baby foods to **[REFERENT CHILD]** more often? **MULTIPLE CODING POSSIBLE**

कृप्या मुझे बताए आप .... के लिए ज्यादातर पैकेज्ड शिशु अनाज/ शिशु खाद्य क्यों नही देते है ?

		Q322
I cannot afford packaged infant cereal/ baby food	मै पैकेज्ड शिशु अनाज/ शिशु आहार का वहन/ अफोर्ड नही कर सकता है	01
It is too expensive as compared to homemade food	यह घर पर बने आहार की तुलना मे बहुत महंगा है	02
There is no small pack to try it and if my child does not eat it, it will be a waste of money	इसे आजमाने के लिए छोटा पैक नही है और यदि मेरा बच्चा इसे नही खाता, यह पैसे की बर्बादी होगी	03
It's not healthy for my child	यह मेरे बच्चे के लिए स्वास्थ्यदायक नही है	04
My child doesn't like it	मेरा बच्चा इसे पसंद नहीं करता है	06
My child is too young for it	मेरे बच्चा इसके लिए बहुत छोटा है	07
My child is too old for it	मेरा बच्चा इसके लिए बहुत बडा है	08
My child gets digestive problem after eating it	इसे खाने के बाद मेरे बच्चे को पाचक परेशानी हो गयी	09
Homemade food has much more variety and child requires variety	घर पर बने आहार मे ज्यादा वैरायटी होती है और बच्चे को वैरायटी चाहिए	12
It does not provide fat	यह फैट प्रदान नही करता है	13
My mother-in-law/ other family members/ friends are against it	मेरी सास/अन्य परिवार के सदस्य/दोस्त इसके विरोध में हैं	26
The doctor advised me not to feed packaged food to my child	डॉक्टर ने मेरे बच्चे को पैकेज्ड आहार नही खिलाने की सलाह दी थी	16
Do not have knowledge about all benefits it provides	इसके द्वारा दिये जाने वाले सभी फायदों के बारे में नहीं जानती	17
It cannot be prepared and kept for a few hours like home food, it has to be eaten immediately	इसे घर पर बने आहार की तरह बनाकर कुछ घंटो के लिए नही रखा जा सकता, इसे तुरंत खाना होता है	18
It is raw/ it is not made by boiling or cooking the food	यह कच्चा है/ यह खाना को पकाने या उबालने द्वारा नही बनाया जाता	19
It may contain preservatives which are harmful	इसमे प्रतिरक्षक/प्रिजर्वेटिव हो सकते है जो हानिकारक है	20
The child will not develop habit of eating homemade food for later in life	जीवन मे बाद के लिए बच्चे मे घर पर बने आहार को खाने की आदत विकसित नही होगी	22
It is very sticky and sticks into the mouth of the child	यह बहुत चिपचिपा है और बच्चे के मुहं मे चिपकता है	24
I feel guilty when I give packaged food to my child	मेरे बच्चे को पैकेज्ड आहार देते समय मै दोषी महसूस करती हूँ	25
Not available in the places where I go for shopping	उस जगह पर उपलब्ध नही जहां से मै खरीदारी करने के लिए जाती हूँ	05
Any other (please specify) ______________	कोई अन्य (कृप्या स्पष्ट करे)	
Any other (please specify) ______________	कोई अन्य (कृप्या स्पष्ट करे)	
➔ **GO TO Q324**		

1411-30

ASK Q323 IF PACKAGED INFANT CEREAL (155) NOT CODED IN Q301 AND '2' CODED IN Q321

Q323 **SHOWCARD Q323** You said that you never bought packaged infant cereals/ baby foods for **[REFERENT CHILD]**. May I know the reason why not? **MULTIPLE CODING POSSIBLE**
आपने कहा कि आपने .... के लिए कभी भी पैकेज्ड शिशु अनाज/ शिशु आहार को नही खरीदाहै । क्या मै कारण जान सकता हूँ क्यों नही ?

		Q323
I cannot afford packaged infant cereal/ baby food	मै पैकेज्ड शिशु अनाज/ शिशु आहार का वहन/ अफोर्ड नही कर सकता है	01
It is too expensive as compared to homemade food	यह घर पर बने आहार की तुलना मे बहुत महंगा है	02
There is no small pack to try it and if my child does not eat it, it will be a waste of money	इसे आजमाने के लिए छोटा पैक नही है और यदि मेरा बच्चा इसे नही खाता, यह पैसे की बर्बादी होगी	03
It's not healthy for my child	यह मेरे बच्चे के लिए स्वास्थ्यदायक नही है	04
My child doesn't like it	मेरा बच्चा इसे पसंद नहीं करता है	06
My child is too young for it	मेरे बच्चा इसके लिए बहुत छोटा है	07
My child is too old for it	मेरा बच्चा इसके लिए बहुत बडा है	08
My child gets digestive problem after eating it	इसे खाने के बाद मेरे बच्चे को पाचक परेशानी हो गयी	09
Homemade food has much more variety and child requires variety	घर पर बने आहार मे ज्यादा वैरायटी होती है और बच्चे को वैरायटी चाहिए	12
It does not provide fat	यह फैट प्रदान नही करता है	13
My mother-in-law/ other family members/ friends are against it	मेरी सास/अन्य परिवार के सदस्य/दोस्त इसके विरोध में हैं	26
The doctor advised me not to feed packaged food to my child	डॉक्टर ने मेरे बच्चे को पैकेज्ड आहार नही खिलाने की सलाह दी थी	16
Do not have knowledge about all benefits it provides	इसके द्वारा दिये जाने वाले सभी फायदों के बारे में नहीं जानती	17
It cannot be prepared and kept for a few hours like home food, it has to be eaten immediately	इसे घर पर बने आहार की तरह बनाकर कुछ घंटो के लिए नही रखा जा सकता, इसे तुरंत खाना होता है	18
It is raw/ it is not made by boiling or cooking the food	यह कच्चा है/ यह खाना को पकाने या उबालने द्वारा नही बनाया जाता	19
It may contain preservatives which are harmful	इसमे प्रतिरक्षक/प्रिजर्वेटिव हो सकते है जो हानिकारक है	20
The child will not develop habit of eating homemade food for later in life	जीवन मे बाद के लिए बच्चे मे घर पर बने आहार को खाने की आदत विकसित नही होगी	22
It is very sticky and sticks into the mouth of the child	यह बहुत चिपचिपा है और बच्चे के मुहं मे चिपकता है	24
I feel guilty when I give packaged food to my child	मेरे बच्चे को पैकेज्ड आहार देते समय मै दोषी महसूस करती हूँ	25
Not available in the places where I go for shopping	उस जगह पर उपलब्ध नही जहां से मै खरीदारी करने के लिए जाती हूँ	05
Any other (please specify) ______________	कोई अन्य (कृप्या स्पष्ट करे)	
Any other (please specify) ______________	कोई अन्य (कृप्या स्पष्ट करे)	

1433-52

ASK ALL

Q324 Please tell me does your household receive packaged infant cereals/ baby foods from social programs? **SINGLE CODING ONLY**
कृप्या मुझे बताए क्या आपका परिवार किसी समाजिक प्रोग्राम से पैकेज्ड शिशु अनाज/ शिशु आहार को प्राप्त करता है ?

There are various food related social programs run by government or any social organization. Under these programs, food is provided to a household directly or indirectly generally either at no cost or at subsidized cost. Some examples of such programs are ration programs, school feeding programs, cash transfers for food etc.

सरकार या अन्य समाजिक संगठनो द्वारा विभिन्न खाद्य संबंधित समाजिक कार्यक्रम चलाए जा रहे है। इन प्रोग्राम के अंतर्गत खाना सीधे एक परिवार को या बिना किसी लागत या कम की गयी कीमत पर परिवारो को दिया जाता है। ऐसे प्रोग्रामो के कुछ कुछ उदाहरण है राशन प्रोग्राम, स्कूल आहार प्रोग्राम, आहार के लिए नगद हस्तांतरण इत्यादि।

Yes हाँ	1	**→ GO TO Q325**
No नही	2	**→ GO TO Q400**

1363

ASK Q325 IF '1'/'YES' CODED IN Q324

Q325 From which program did you receive the packaged infant cereals/ baby foods you gave to **[REFERENT CHILD]? RECORD VERBATIM**

किस प्रोग्राम से आपने .... को देने के लिए पैकेज्ड शिशु अनाज/ शिशु आहार को प्राप्त किया था ?

1364-72

PRICE SENSITIVITY SECTION

TO NON BUYERS ASK ONLY APPROACH 2

FOR CURRENT BUYERS, SELECT FROM RANDOM LIST WHETHER APPROACH 1 OR APPROACH 2 COMES FIRST

Q400 SELECT APPROACH THAT COMES FIRST FROM RANDOM LIST

GRID ONLY FOR CURRENT BUYERS				
**PRICING APPROACH PANELS (SINGLE CODING ONLY) See number on RANDOM LIST.**				
	**ASKED FIRST**	**ASKED SECOND**		
**Panel 1**	Approach 1 (start at Q401)	Approach 2 (start at Q420)	1	1477
**Panel 2**	Approach 2 (start at Q420)	Approach 1 (start at Q401)	2	

APPROACH 1

ASK Q401-Q415 TO CURRENT BUYERS

DETERMINE CURRENT BRAND
Brand (Q307a): ____________________
Pack Type (Pack type of brand coded in Q307a): ___________________
Pack Size (Pack size of brand coded in Q307a): ___________________

Q401 You mentioned that you serve **[BRAND, PACK TYPE AND PACK SIZE]** most often to your child. Please tell me at what price do buy each pack of this brand, pack type and size? **RECORD VERBATIM WITH LEADING ZEROES**
आपने बताया कि आपके बच्चे के लिए आप ज्यादातर .... सर्व करती है। कृप्या मुझे बताए आप इस ब्रांड, पैक प्रकार और साइज के प्रत्येक पैक को किस कीमत पर खरीदती है ?

**PRICE OF MOST OFTEN USED PACK (in Rs.)**					1478-81

Q402 How many packs of **[BRAND, PACK TYPE AND PACK SIZE]** do you buy in a usual shopping trip? **RECORD VERBATIM WITH LEADING ZEORES**
.... के कितने पैक्स को आप सामान्य खरीदारी मे खरीदती है ?

**NUMBER OF PACKS PER SHOPPING TRIP**			1482-83

Q403 How often do you make such shopping trips? **RECORD VERBATIM WITH LEADING ZEROES FOR NUMBER OF DAYS BETWEEN SHOPPING TRIPS**
आप ऐसी खरीदारी कितनी बार करती है ?

**NUMBER OF DAYS BETWEEN SHOPPING TRIPS**			1484-85

Q404 Select % DISCOUNT from RANDOM LIST

**Discount in RANDOM LIST**	**SINGLE CODE**

20%	1
40%	2
60%	3
80%	4

1486

Q405 COMPUTE DISCOUNTED PRICE:

DISCOUNT SELECTED	1	2	3	4	
Brand _________	DISCOUNT - 20%	DISCOUNT - 40%	DISCOUNT - 60%	DISCOUNT - 80%	DISCOUNTED PRICE
Price Paid per Pack (Q401) _________	___ x 0.8	___ x 0.6	___ x 0.4	___ x 0.2	

1511-16

EXAMPLE FOR COMPUTING DISCOUNTED PRICE:
Brand – Cerelac – 1000 gram
Price paid per pack – Rs. 365
Discount Selected – '2'/ Discount 40%

DISCOUNTED PRICE = 365 x 0.6
= Rs. 219

DISCOUNT SELECTED	1	(2)	3	4	
Brand Cerelac 1000 gm	DISCOUNT - 20%	DISCOUNT - 40%	DISCOUNT - 60%	DISCOUNT - 80%	DISCOUNTED PRICE
Price Paid per Pack (Q401) Rs. 365	___ x 0.8	___ x 0.6	___ x 0.4	___ x 0.2	Rs. 219

ASK Q406- 415 FOR DISCOUNTED PRICE

SAY: We are interested in finding out how much packaged infant cereal/ baby food you would buy at a lower price. This lower price would be a permanently lower price and not just a temporary promotion price. At this price, product is going to be regularly available in the market
हम यह पता लगाने मे दिलचस्प है कि आप कम कीमत पर कितना पैकेज्ड शिशु अनाज/ शिशु आहार खरीदेंगी। कम कीमत स्थायी कम कीमत होगी और एक अस्थायी प्रोमोशन कीमत नही। इस कीमत पर, प्रोडक्ट बाजार मे नियमित रूप से उपलब्ध होगा।

Q406 **SHOWCARD 406** please give me your opinion on value for money of this packaged infant cereal/ baby food if one package of this was available at **[DISCOUNTED PRICE]**? **SINGLE CODING ONLY**
कृप्या मुझे इस पैकेज्ड शिशु अनाज/ शिशु आहार की पैसे के लिए वसुली पर अपनी राय दे यदि इसका एक पैकेज .... पर उपलब्ध था?

Excellent	सर्वोत्तम	05
Very good	बहुत अच्छा	04
Good	अच्छा	03
Fair	ठीक	02
Poor	खराब	01
Don't know **(DO NOT SHOW)**	पता नही	98

1517-18

Q407 **SHOWCARD 407** please give tell me how willing are you to buy this packaged infant cereal/ baby food in future if one package of this was available at **[DISCOUNTED PRICE]**? **SINGLE CODING ONLY**
कृप्या मुझे बताए आपकी भविष्य मे इस पैकेज्ड शिशु अनाज/ शिशु आहार को खरीदने की कितनी संभावना है यदि इसका एक पैकेज .... पर उपलब्ध हो?

Will definitely buy	निश्चित रूप से खरीदेंगें	05	→ GO TO 408
Will probably buy	शायद खरीदेंगें	04	
May or may not buy	खरीद भी सकते है या नही भी	03	
Will probably not buy	शायद नही खरीदेंगें	02	
Will definitely not buy	निश्चित रूप से नही खरीदेंगें	01	→ GO TO 501 OR APP 2
Don't know **(DO NOT SHOW)**	पता नही	98	→ GO TO 408

1519-20

ASK Q408 IF CODED '2'/'3'/'4'/'5'/'98' CODED IN Q407

Q408 How many packs of **[BRAND NAME, PACK TYPE AND PACK SIZE]** would you buy in a usual shopping trip if the price of a package was **[DISCOUNTED PRICE]**? **RECORD VERBATIM WITH LEADING ZEROES**
.... के कितने पैक्स को आप सामान्य खरीदारी मे खरीदेंगी यदि पैकेज की कीमत . ... हो?

NUMBER OF PACKS PER SHOPPING TRIP			1521-22

Q409 How often would you make such shopping trips? **RECORD VERBATIM WITH LEADING ZEROES FOR NUMBER OF DAYS BETWEEN SHOPPING TRIPS**
आप ऐसी खरीदारी कितनी बार करेंगी ?

NUMBER OF DAYS BETWEEN SHOPPING TRIPS			1523-24

Q410 Will you serve more packaged infant cereal/ baby food as a result of this lower price or will consumption remain the same? **SINGLE CODING ONLY**
क्या आप कम कीमत के परिणाम स्वरूप ज्यादा पैकेज्ड शिशु अनाज/ शिशु आहार को सर्व करेंगी या खपत समान रहेगी ?

Yes, will serve more packaged infant cereal	हाँ, ज्यादा पैकेज्ड शिशु अनाज सर्व करेंगें	01	→ **GO TO 411**
No, will NOT serve more packaged infant cereal/ Consumption will remain the same	नही, पैकेज्ड शिशु अनाज ज्यादा सर्व नही करेंगें / खपत समान रहेगी	02	→ **GO TO 501 OR APP 2**
Don't know **(DO NOT SHOW)**	पता नही	98	→ **GO TO 411**

1525-26

ASK Q411 IF CODED '1' IN Q410

Q411 **SHOWCARD Q411** If you will serve more packaged infant cereal/ baby food that you currently do, who would consume the ADDITIONAL packaged infant cereal/ baby food? **SINGLE CODING ONLY**
यदि आप वर्तमान से ज्यादा पैकेज्ड शिशु अनाज/ शिशु आहार को सर्व करेंगी, तो कौन अतिरिक्त पैकेज्ड शिशु अनाज/ शिशु आहार को खाएगा ?

Equally distributed among adults and children	व्यस्को और बच्चो के बीच बराबर बांटा था	01	→ **GO TO 412**
Only adults	केवल व्यस्क	02	→ **GO TO 501 OR APP 2**
Mainly adults	मुख्य रूप से व्यस्क	03	
Mainly children	मुख्य रूप से बच्चे	04	→ **GO TO 412**
Only child/ children	केवल बच्चा/ बच्चे	05	
Don't know **(DO NOT SHOW)**	पता नही	98	→ **GO TO 501 OR APP 2**

1527-28

ASK Q412 IF '1'/'4'/'5' CODED IN Q411

Q412 **SHOWCARD Q412** How will the ADDITIONAL packaged infant cereal/ baby food be distributed among the children? **SINGLE CODING ONLY**
अतिरिक्त पैकेज्ड शिशु अनाज/ शिशु आहार को बच्चो के बीच कैसे बटवारा किया जाएगा ?

IF ONLY 1 CHILD IN THE FAMILY, AUTOCODE '2'

Equally distributed among all children	सभी बच्चो के बीच बराबर बांटा था	01	→ **GO TO 413**
Only **[REFERENT CHILD]**	केवल	02	
Mainly **[REFERENT CHILD]**	मुख्य रूप से	03	
Mainly the other children	मुख्य रूप से अन्य बच्चे	04	
Only the other children	केवल अन्य बच्चे	05	→ **GO TO 501 OR APP 2**
Don't know **(DO NOT SHOW)**	पता नही	98	→ **GO TO 413**

1529-30

ASK Q413 IF '1'/'2'/'3'/'4'/'98' CODED IN Q412

Q413 Would you feed less of other food and drinks for **[REFERENT CHILD]**? **SINGLE CODING ONLY**
क्या आप .... के अन्य आहार और ड्रिंक्स कम खिलायेंगें/ पिलायेंगें ?

Yes हाँ	1	→ **GO TO 414**
No नही	2	→ **GO TO 501 OR APP 2**
Don't know **(DO NOT SHOW)** पता नही	98	

1531

ASK Q414 IF '1' / 'YES' CODED IN Q413

Q414 **SHOWCARD Q414** What kind of food and drinks would you feed less to **[REFERENT CHILD]**? **MULTIPLE CODING POSSIBLE**
आप .... के लिए किस प्रकार के आहार और ड्रिंक्स को कम खिलायेंगें/ पिलायेंगें ?

**Breast Milk**	माँ का दूध	01
Cereals like **Daliya/ Khichdi/ Mixed Grains/ Sewiya**	सीरियल्स जैसे दलिया/खिचड़ी/मिले अनाज/सेवईंया	02
Cereals like **Ragi/ Barley/ Bajra/ Maize/ Corn**	सीरियल्स जैसे रागी/जौं/बाजरा/मक्का/दाना	03
**Oats/ Corn flakes/ Rice flakes**	ओट्स/ कोर्नफ्लेक्स/ चावल के फ्लेक्स	04

**Rice/ Rice based dishes**	चावल/ चावल से बने व्यंजन	05
**Packaged Infant Cereals**	पैकेज्ड इनफेंट सीरियल्स/ बेबी फूड	06
**Legumes** like pulses, chana etc.	फली जैसे दालें, चना आदि	07
**Roti, Paratha, Bread, Dosa, Poori etc.**	रोटी, पराठा, ब्रेड, डोसा, पूरी आदि	08
**Packaged Powdered Milk**	पैकेज्ड पाउडर्ड दूध	09
**Homemade Snacks** like poha, semolina/ suji/ rawa, halwa, upma, idli, puffed rice, Idiyappam, sattu, chattuah, sabudana etc.	घर में बने स्नेक्स जैसे पोहा, सेमोलिना/सूजी/रवा, हलवा, उपमा, इडली, फूले चावल, इडियापन, सत्तू, छतूआ, साबुदाना आदि	10
**Cow milk/ buffalo milk (packaged or loose)**	गाय का दूध/भैंस का दूध (पैकेज्ड या खुला)	11
**Milk products** like curd, raita, lassi/ chaach, cream, tea, coffee, paneer, khoya, shakes, ice cream etc	दूध के प्रोडक्ट्स जैसे दही, रायता, लस्सी/छाछ, क्रीम, चाय, कॉफी, पनीर, खोवा, शेक्स, आईसक्रीम आदि.	12
**Non vegetarian foods and non veg soups**	माँसाहारी खाने और माँसाहारी सूप्स	13
**Infant formulas**	इनफेंट फार्मूला	14
**Fruits/ Fruit Juices/ Neembu pani**	फल/ फलों के जूस/ नींबू पानी	15
**Packaged snacks/ foods** like biscuits, chips, soft drinks, confectionary items, namkeens, milk additives likes Horlicks etc.	पैकेज्ड स्नेक्स/फूड्स जैसे बिस्कुट्स, चिप्स, सॉफ्ट ड्रिंक्स, कॉन्फेक्शनरी सामग्रियाँ, नमकींस, दूध एडीटिव्स जैसे हॉरलिक्स आदि	16
**Vegetables and vegetable soups**	सब्जियाँ और सब्जियों के सूप्स	17
**Any other** ________________________<br>कोई अन्य		
**Any other** ________________________<br>कोई अन्य		
Don't know/Don't remember **(DON'T SHOW/ READ)**<br>पता नहीं/याद नहीं		98

1532-47

Q415 Please tell me whether you would buy less of other brands of packaged infant cereals/ baby food that you buy regularly? **MULTIPLE CODING POSSIBLE**
कृप्या मुझे बताए क्या आप पैकेज्ड शिशु अनाज/ शिशु आहार के अन्य ब्रांडस को कम खरीदेंगें जो कि आप नियमित रुप से खरीदते है?

	**BRANDS CODED IN Q304**	
Brand 1 **(Specify Name)**<br>ब्रांड 1 (नाम स्पष्ट करे) ____________	1	1548-50
Brand 2 **(Specify Name)**<br>ब्रांड 2 (नाम स्पष्ट करे)____________	2	1551-53
Brand 3 **(Specify Name)**<br>ब्रांड 3 (नाम स्पष्ट करे) ____________	3	1554-56
Brand 4 **(Specify Name)**<br>ब्रांड 4 (नाम स्पष्ट करे) ____________	4	1557-59
Brand 5 **(Specify Name)**<br>ब्रांड 5 (नाम स्पष्ट करे) ____________	5	1560-62
**Will not reduce for any brand**<br>किसी ब्रांड के लिए कम नही करेंगें	9	1563
**→ GO TO 501 OR APP 2**		

APPROACH 2- ASK ALL

SAY: Suppose you were eligible for a program allowing you, as the mother of a child aged 6-23 months old, to buy packaged infant cereal/ baby food at a reduced price. Each pack of this special infant cereal for children 6-23 months of age would be sufficient to prepare 1 serving of cereals for **[REFERENT CHILD]**. The required daily amount of this infant cereal is 2 servings per day i.e. 2 packs per day.

कहेः कल्पना करे आप एक प्रोग्राम के लिए योग्य है जो आपको 6–23 महीनो की उम्र के बच्चे की माँ के रूप मे, पैकेज्ड शिशु अनाज/ शिशु आहार एक कम की गयी कीमत पर खरीदने की अनुमति देता है। इस विशेष शिशु अनाज का प्रत्येक पैक 6–23 महीनो की उम्र के बच्चे के लिए प्रति दिन अनाज की 1 सर्विंग बनाने के लिए पर्याप्त होगा। इस शिशु अनाज की प्रति दिन की ज़रूरी मात्रा प्रति दिन 2 सर्विंग है मतलब प्रति दिन 2 पैक।

Please note that this product is not a substitute for breastfeeding and that **[REFERENT CHILD]** will still require other food items along with this.
कृपया ध्यान रहे कि यह प्रोडक्ट स्तनपान का विकल्प नहीं है और .... को इसके साथ अन्य खाने की सामग्रियों की जरूरत होगी।

This special price would be a permanently lower price and not just a temporary promotion price. At this price, the product is going to be regularly available in the market.
यह विशेष कीमत स्थायी रूप से कम कीमत होगी और केवल एक अस्थायी कीमत नही होगी। इस कीमत पर, प्रोडक्ट नियमित रूप से बाजार मे उपलब्ध होगा।

According to the programme (child 6-23 months of age) should be fed with 2 packages per day until she/ he reaches the age of 2 years. Please note that this packaged infant cereal/ baby food is for children 6 months to less than 2 years old only and is not advisable to be served to others not within this age range.
प्रोग्राम के अनुसार 6–23 महीने की उम्र के बच्चे को प्रतिदिन 2 पैकेज खिलाना चाहिए जब तक वह 2 वर्ष की उम्र तक नही पहुंचे। कृप्या याद रखें कि यह पैकेज्ड शिशु अनाज/ शिशु आहार केवल 6 महीनो से 2 वर्ष से कम उम्र के बच्चे के लिए है और इस रेंज के बाहर के अन्य को सर्व करने के लिए नही है।

We are interested in finding out how much of this packaged infant cereal/ baby food you would buy.
हम यह पता लगाने मे दिलचस्प है आप इस पैकेज्ड शिशु अनाज/ शिशु आहार को कितना खरीदेंगें।

EXPOSE CONCEPT CARD AND DEBRANDED PACKS

Q420 Select START_PRICE _2 from RANDOM LIST. START WITH THAT PRICE

SINGLE CODING ONLY					
**P1**<br>**Rs. 14**	**P2**<br>**Rs. 11**	**P3**<br>**Rs. 8**	**P4**<br>**Rs. 5**	**P5**<br>**Rs. 2**	
1	2	3	4	5	1564

Q421 Would you buy this packaged infant cereal/ baby food if the price of 1 package was **[MENTION PRICE]? SINGLE CODING ONLY**
क्या आप इस शिशु अनाज/ शिशु आहार को खरीदेंगें यदि 1 पैकेज की कीमत .... हो ।

Yes हाँ	1	→ GO TO 423
No नही	2	→ GO TO 422
Don't know **(DO NOT SHOW)** पता नही	98	
	1565-66	

Q422 Would you buy this packaged infant cereal/ baby food if the price of 1 package was **[NEXT LOWER PRICE]? If "NO" CONTINUE TO NEXT LOWER PRICE UNTIL LOWEST PRICE. SINGLE CODING ONLY**
क्या आप इस शिशु अनाज/ शिशु आहार को खरीदेंगें यदि 1 पैकेज की कीमत .... हो ।

**CIRCLE THE STARTING PRICE**	1	2	3	4	5		
**= NEXT LOWER PRICE TO START_PRICE_2**	**Rs. 14**	**Rs. 11**	**Rs. 8**	**Rs. 5**	**Rs. 2**		
**CIRCLE THE PRICE AT WHICH RESPONDENT SAID 'YES'**	**1**	**2**	**3**	**4**	**5**	**None - 9**	1567
	**→ GO TO 423**					**→ GO TO 431**	

ASK Q423 FOR PRICE FOR WHICH '1'/'YES' CODED IN Q421 OR Q422 = BUY_PRICE_2

**WRITE "BUY PRICE 2 INTO BOX:**		

1568-69

Q423 Would you feed less of other food and drinks for **[REFERENT CHILD]**? **SINGLE CODING ONLY**
क्या आप .... को अन्य आहार और ड्रिंक्स कम खिलायेंगें/ पिलायेंगें ?

Yes हाँ	1	→ **GO TO 424**
No नही	2	→ **GO TO 425**
Don't know **(DO NOT SHOW)** पता नही	98	

1570-71

ASK Q424 IF '1' CODED IN Q423

Q424 **SHOWCARD Q424** What kind of food and drinks would you feed less to **[REFERENT CHILD]**? **MULTIPLE CODING POSSIBLE**
आप .... को किस प्रकार का आहार और ड्रिंक्स कम खिलायेंगें/ पिलायेंगें ?

**Breast Milk**	माँ का दूध	01
Cereals like **Daliya/ Khichdi/ Mixed Grains/ Sewiya**	सीरियल्स जैसे दलिया/खिचड़ी/मिले अनाज/सेवईंया	02
Cereals like **Ragi/ Barley/ Bajra/ Maize/ Corn**	सीरियल्स जैसे रागी/जौं/बाजरा/मक्का/दाना	03
**Oats/ Corn flakes/ Rice flakes**	ओट्स/ कोर्नफ्लेक्स/ चावल के फ्लेक्स	04
**Rice/ Rice based dishes**	चावल/ चावल से बने व्यंजन	05
**Packaged Infant Cereals**	पैकेज्ड इनफेंट सीरियल्स/ बेबी फूड	06
**Legumes** like pulses, chana etc.	फली जैसे दालें, चना आदि	07
**Roti, Paratha, Bread, Dosa, Poori etc.**	रोटी, पराठा, ब्रेड, डोसा, पूरी आदि	08
**Packaged Powdered Milk**	पैकेज्ड पाउडर्ड दूध	09
**Homemade Snacks** like poha, semolina/ suji/ rawa, halwa, upma, idli, puffed rice, Idiyappam, sattu, chattuah, sabudana etc.	घर में बने स्नेक्स जैसे पोहा, सेमोलिना/सूजी/रवा, हलवा, उपमा, इडली, फूले चावल, इडियापन, सत्तू, छतूआ, साबुदाना आदि	10
**Cow milk/ buffalo milk (packaged or loose)**	गाय का दूध/भैंस का दूध (पैकेज्ड या खुला)	11
**Milk products** like curd, raita, lassi/ chaach, cream, tea, coffee, paneer, khoya, shakes, ice cream etc	दूध के प्रोडक्ट्स जैसे दही, रायता, लस्सी/छाछ, क्रीम, चाय, कॉफी, पनीर, खोवा, शेक्स, आईसक्रीम आदि.	12
**Non vegetarian foods and non veg soups**	माँसाहारी खाने और माँसाहारी सूप्स	13
**Infant formulas**	इनफेंट फार्मूला	14
**Fruits/ Fruit Juices/ Neembu pani**	फल/ फलों के जूस/ नींबू पानी	15
**Packaged snacks/ foods** like biscuits, chips, soft drinks, confectionary items, namkeens, milk additives likes Horlicks etc.	पैकेज्ड स्नेक्स/फूड्स जैसे बिस्कुट्स, चिप्स, सॉफ्ट ड्रिंक्स, कॉन्फेक्शनरी सामग्रियाँ, नमकींस, दूध एडीटिव्स जैसे हॉरलिक्स आदि	16
**Vegetables and vegetable soups**	सब्जियाँ और सब्जियों के सूप्स	17
**Any other** ________________________ कोई अन्य		
**Any other** ________________________ कोई अन्य		
Don't know/Don't remember **(DON'T SHOW/ READ)** पता नहीं/याद नहीं		98

1572-87

Q425 **SHOWCARD Q425** Who in your household would consume the packaged infant cereal/ baby food? **SINGLE CODING ONLY**
आपके परिवार मे कौन पैकेज्ड शिशु अनाज/ शिशु आहार को खायेंगा ?

Equally distributed among adults and children	व्यस्को और बच्चो के बीच बराबर बांटना	01	→ **GO TO 426**
Only adults	केवल व्यस्क	02	→ **GO TO 427**
Mainly adults	मुख्य रूप से व्यस्क	03	
Mainly children	मुख्य रूप से बच्चे	04	→ **GO TO 426**
Only child/ children	केवल बच्चा/ बच्चे	05	
Don't know **(DO NOT SHOW)**	पता नही	98	→ **GO TO 427**

1611-12

ASK Q426 IF '1'/'4'/'5' CODED IN Q425

Q426 **SHOWCARD Q426** How will the packaged infant cereal/ baby food be distributed among the children? **SINGLE CODING ONLY**
बच्चो के बीच पैकेज्ड शिशु अनाज/ शिशु आहार को कैसे बटवारा किया जाएगा ?

IF ONLY 1 CHILD IN THE FAMILY, AUTOCODE '2'

Equally distributed among all children	सभी बच्चो के बीच बराबर बांटना	01

Only **[REFERENT CHILD]**	केवल	02
Mainly **[REFERENT CHILD]**	मुख्य रूप से	03
Mainly the other children	मुख्य रूप से अन्य बच्चे	04
Only the other children	केवल अन्य बच्चे	05
Don't know **(DO NOT SHOW)**	पता नही	98

1613-14

ASK ALL

Q427 **SHOWCARD Q427** please give me your opinion on value for money of this packaged infant cereal/ baby food if one package of this was available at **(PRICE_BUY_2)**? **SINGLE CODING ONLY**
कृप्या मुझे इस पैकेज्ड शिशु अनाज/ शिशु आहार की पैसे के लिए वसुली पर अपनी राय दे यदि इसका एक पैकेज .... पर उपलब्ध था ?

Excellent	सर्वोत्तम	05
Very good	बहुत अच्छा	04
Good	अच्छा	03
Fair	ठीक	02
Poor	खराब	01
Don't know **(DO NOT SHOW)**	पता नही	98

1615-16

Q428 **SHOWCARD 428** please tell me how willing are you to buy this packaged infant cereal/ baby food in future if one package of this was available at **(PRICE_BUY_2)**? **SINGLE CODING ONLY**
कृप्या मुझे बताए आपकी भविष्य मे इस पैकेज्ड शिशु अनाज/ शिशु आहार को खरीदने की कितनी संभावना है यदि इसका एक पैकेज .... पर उपलब्ध हो ?

Will definitely buy	निश्चित रूप से खरीदेंगें	05	**→ GO TO 429**
Will probably buy	शायद खरीदेंगें	04	
May or may not buy	खरीद भी सकते है या नही भी	03	
Will probably not buy	शायद नही खरीदेंगें	02	
Will definitely not buy	निश्चित रूप से नही खरीदेंगें	01	**→ GO TO APPROACH 1 IF CURRENT BUYER AND APPROACH 1 NOT YET DONE - ELSE GO TO Q501**
Don't know **(DO NOT SHOW)**	पता नही	98	

1617-18

ASK Q429 IF CODED '5'/'4'/'3'/'2' IN Q428

Q429 How many packs of **[CONTROL PACK]** would you buy in a week? **RECORD VERBATIM WITH LEADING ZEROES**
आप एक सप्ताह मे .... के कितने पैक्स को खरीदेंगें ?

**NUMBER OF PACKS PER WEEK**		

1619-20

Q430 How many servings of **[CONTROL PACK]** would you give to **[REFERENT CHILD]** in a week? **RECORD VERBATIM WITH LEADING ZEROES**
आप एक सप्ताह मे .... को .... की कितनी सर्विंग देंगें ?

**NUMBER OF SERVINGS PER WEEK**		

1621-22

ASK Q431 IF CODED '9' IN Q422

Q431 Would you feed **[CONTROL PACK]** to **[REFERENT CHILD]** if you would receive **[CONTROL PACK]** for **FREE**? **SINGLE CODING ONLY**
अगर आप को .... मुफत मे मिले तो क्या आप .... को यह देंगें ?

Yes<br>हाँ	01
No<br>नही	02
Don't know **(DO NOT SHOW)**<br>पता नही	98

1673-74

→ GO TO APPROACH 1 IF CURRENT BUYER AND APPROACH 1 NOT YET DONE

→ **ELSE GO Q501**

ATTITUDES SECTION

Q501 Did you receive any advice on which type of foods/ drinks you should give to your child? **SINGLE CODING ONLY**
क्या आपने कोई सलाह प्राप्त की थी कि आपके बच्चे के लिए किस प्रकार का आहार/ ड्रिंक्स दिए जाने चाहिए ?

Yes<br>हाँ	1
No<br>नही	2

1623

Q502 **SHOWCARD Q502** Do you remember who gave you the advice? **MULTIPLE CODING POSSIBLE**
क्या आपको याद है सलाह किसने दी थी ?

A paediatrician	एक बाल विशेषज्ञ	01
Another type of health professional	स्वास्थ्य पेशेवर का कोई प्रकार	02
A relative (own mom, aunt, mother-in-law) or friend	एक रिश्तेदार या दोस्त	03
Information from TV, radio, newspaper or other media	टीवी, रेडियो, अखबार, या अन्य मीडिया से जानकारी	04
Others (Please specify _____________)	अन्य (कृप्या स्पष्ट करे ....)	
Others (Please specify _____________)	अन्य (कृप्या स्पष्ट करे ....)	
Don't know/Don't remember **(DON'T SHOW/ READ)**	पता नही/ याद नही	98

1624-31

Q504 Are there any foods containing iron? **SINGLE CODING ONLY**
क्या किसी फूड/आहार में आयरन होता है ?

Yes हाँ	01
No नही	02
Don't know/Don't remember **(DON'T SHOW/ READ)**<br>पता नही/ याद नही	98

1647-48

ASK Q505/ Q506 IF CODED '1' IN Q504

Q505 **SHOWCARD Q505** Do you know which foods and drinks are rich in iron? **MULTIPLE CODING POSSIBLE**
क्या आपको पता है कौन से खाद्य और पेय आयरन मे समृद्ध है ?

**Breast Milk**	माँ का दूध	01
Cereals like **Daliya/ Khichdi/ Mixed Grains/ Sewiya**	सीरियल्स जैसे दलिया/खिचड़ी/मिले अनाज/सेवईंया	02
Cereals like **Ragi/ Barley/ Bajra/ Maize/ Corn**	सीरियल्स जैसे रागी/जौं/बाजरा/मक्का/दाना	03
**Oats/ Corn flakes/ Rice flakes**	ओट्स/ कोर्नफ्लेक्स/ चावल के फ्लेक्स	04
**Rice/ Rice based dishes**	चावल/ चावल से बने व्यंजन	05
**Packaged Infant Cereals**	पैकेज्ड इनफेंट सीरियल्स/ बेबी फूड	06
**Legumes** like pulses, chana etc.	फली जैसे दालें, चना आदि	07
**Roti, Paratha, Bread, Dosa, Poori etc.**	रोटी, पराठा, ब्रेड, डोसा, पूरी आदि	08
**Packaged Powdered Milk**	पैकेज्ड पाउडर्ड दूध	09
**Homemade Snacks** like poha, semolina/ suji/ rawa, halwa, upma, idli, puffed rice, Idiyappam, sattu, chattuah, sabudana etc.	घर में बने स्नेक्स जैसे पोहा, सेमोलिना/सूजी/रवा, हलवा, उपमा, इडली, फूले चावल, इडियापन, सत्तू, छत्तूआ, साबुदाना आदि	10
**Cow milk/ buffalo milk (packaged or loose)**	गाय का दूध/भैंस का दूध (पैकेज्ड या खुला)	11
**Milk products** like curd, raita, lassi/ chaach, cream, tea, coffee, paneer, khoya, shakes, ice cream etc	दूध के प्रोडक्ट्स जैसे दही, रायता, लस्सी/छाछ, क्रीम, चाय, कॉफी, पनीर, खोवा, शेक्स, आईसक्रीम आदि.	12
**Non vegetarian foods and non veg soups**	माँसाहारी खाने और माँसाहारी सूप्स	13
**Infant formulas**	इनफेंट फार्मूला	14
**Fruits/ Fruit Juices/ Neembu pani**	फल/ फलों के जूस/ नींबू पानी	15
**Packaged snacks/ foods** like biscuits, chips, soft drinks, confectionary items, namkeens, milk additives likes Horlicks etc.	पैकेज्ड स्नेक्स/फूड्स जैसे बिस्कुट्स, चिप्स, सॉफ्ट ड्रिंक्स, कॉन्फेक्शनरी सामग्रियाँ, नमकींस, दूध एडीटिव्स जैसे हॉरलिक्स आदि	16
**Vegetables and vegetable soups**	सब्जियाँ और सब्जियों के सूप्स	17
**Any other** ________________________ कोई अन्य		
**Any other** ________________________ कोई अन्य		
Don't know/Don't remember **(DON'T SHOW/ READ)** पता नहीं/याद नहीं		98

1649-64

Q506 **SHOWCARD Q506** What are the health consequences of too little iron in foods and drinks? **MULTIPLE CODING POSSIBLE**
खाने और पीने मे बहुत थोडे आयरन के स्वास्थ्य परिणाम क्या है ?

Impact on eyesight	आँखों की दृष्टि पर प्रभाव	05
Anemia/ Blood deficiency	एनिमिया/ रक्त की कमी	01
Lethargy/ Weakness	सुस्ती/ क्मजोरी	02
Skin Related problems like allergy, rashes	त्वचा से सम्बन्धित समस्यायें जैसे एलर्जी, चकत्ते/ रैश	06
Hair related problems	बालों से सम्बन्धित समस्यायें	07
Irritability	चिडचिडापन	03
Problems in digestion	पाचन की समस्यायें	08
Impact on bones strength	हड्डियों की मजबूती पर प्रभाव	09
Mental development	मानसिक विकास	04
Others, please specify: ________________	अन्य, कृप्या स्पष्ट करे	
Others, please specify: ________________	अन्य, कृप्या स्पष्ट करे	
Don't know/Don't remember **(DON'T SHOW/ READ)**	पता नही/ याद नही	98

1665-72

ASK Q510-514 IF PACKAGED INFANT CEREAL CODED IN Q301 (SERVED IN PAST 3 DAYS)

Q510 Do you serve packaged infant cereals/ baby foods that contain added vitamins and minerals? **SINGLE CODING ONLY**
क्या आप पैकेज्ड शिशु अनाज/ शिशु आहार सर्व करती है जो कि विटामिन और मिनरल युक्त होते है ?

Yes<br>हाँ	01
No<br>नही	02
Don't know/Don't remember **(DON'T SHOW/ READ)**<br>पता नही/ याद नही	98

1750-51

Q511 Until what age do you plan to provide packaged infant cereals/ baby foods to your child? **IF DON'T KNOW; RECORD 98**

किस उम्र तक आप आपके बच्चे के लिए पैकेज्ड शिशु अनाज/ शिशु आहार प्रदान करने की योजना बना रही है?

**Maximum age of child (in years)**		

1752-53

Q512 **SHOWCARD Q512** Did you receive any advice on giving packaged infant cereals/ baby foods to **[REFERENT CHILD]**? **SINGLE CODING ONLY**

क्या आपने .... के लिए पैकेज्ड शिशु अनाज/ शिशु आहार देने पर कोई सलाह प्राप्त की थी?

Yes, I have received advice **NOT** to give packaged infant cereals/ baby foods to **[REFERENT CHILD]**	हाँ, मैने .... के लिए पैकेज्ड शिशु अनाज/ शिशु आहार **नही** देने की सलाह प्राप्त की है	01
Yes, I have received advice to give packaged infant cereals/ baby foods to **[REFERENT CHILD]**	हाँ, मैने .... के लिए पैकेज्ड शिशु अनाज/ शिशु आहार देने की सलाह प्राप्त की है	02
No, I have not received any advice	नही, मैने कोई सलाह प्राप्त नही की है	03
Don't know/Don't remember **(DON'T SHOW/ READ)**	पता नही/ याद नही	98

1754-55

Q513 **SHOWCARD Q513** Do you remember who gave you the advice? **MULTIPLE CODING POSSIBLE**
क्या आपको याद है सलाह किसने दी थी?

A paediatrician	एक बाल विशेषज्ञ	01
Another type of health professional	स्वास्थ्य पेशेवर का कोई प्रकार	02
A midwife	एक मिडवाइफ	03
A relative (own mom, aunt, mother-in-law) or friend	एक रिश्तेदार या दोस्त	04
Information from TV, radio, newspaper or other media	टीवी, रेडियो, अख्बार, या अन्य मीडिया से जानकारी	05
Others, please specify: ______________	अन्य, कृप्या स्पष्ट करे	
Don't know/Don't remember **(DON'T SHOW/ READ)**	पता नही/ याद नही	98

1756-65

Q514 What was the age of **[REFERENT CHILD]** when the advice was given? **RECORD IN MONTHS. IF ADVICE WAS GIVEN DURING PREGNANCY PUT "00"**
.... की उम्र क्या थी जब सलाह गयी थी?

**Age of child (IN MONTHS) when advice was given**		

1766-67

CLASSIFICATION SECTION

Q601 How many people are permanently living in this house? Please exclude household help/s if any. **RECORD VERBATIM WITH LEADING ZEROES**
इस परिवार मे स्थायी रुप से कितने लोग रहते है? कृप्या घरेलु सहायको को शामिल नही करे।

**No. of members in HH**		

1768-69

Q602 And how many working members are there in your household? **RECORD VERBATIM WITH LEADING ZEROES**
और आपके परिवार मे कितने कार्यरत सदस्य है?

**No. of working members in HH**		

1770-71

Q603 May we know which of these monthly household income ranges does your household monthly income belongs to? **SINGLE CODING ONLY**
क्या हम जान सकते है इनमे से कौन सा मासिक पारिवारीक आय रेंज मे आपके परिवार की मासिक आय संबंध रखती है ?

Less than Rs. 3,000<br>३००० रूपए से कम	1
Rs. 3,001 to Rs. 5,000	2
Rs. 5,001 to Rs. 10,000	3
Rs. 10,001 to Rs. 20,000	4
Rs. 20,001 to Rs. 30,000	5
Rs. 30,001 to Rs. 40,000	6
Rs. 40,001 to Rs. 50,000	7
More than Rs. 50,000<br>५०,००० रूपए से ज्यादा	8
Not disclosed **(DO NOT SHOW/ READ)**<br>नही बताया	9

1772

Q604 **SHOWCARD Q604** Please tell me which of the following applies to the your house? **SINGLE CODING ONLY**
कृप्या मुझे बताए निम्नलिखित मे से कौन सा आपके घर के लिए लागु होता है ?

Apartment	अपार्टमेंट	1
Bungalow	बंग्लो	2
Independent House	स्वतंत्र मकान	3
Independent Floor	स्वतंत्र फ्लोर	4

1773

Q605 **SHOWCARD Q605** And which of the following applies to the ownership of this house? **SINGLE CODING ONLY**
और निम्नलिखित मे से कौन सा इस घर के लिए स्वामित्व पर लागु होता है ?

Rented	किराए का	1
Owned	स्वामित्व प्राप्त	2

1774

Q606 Do you or any member in your household own any other house? **SINGLE CODING ONLY**
क्या आप या आपके परिवार का कोई सदस्य किसी अन्य घर का स्वामित्व रखता है ?

Q607 Do you or any member in your household own any agricultural land**? SINGLE CODING ONLY**
क्या आप या आपके परिवार का कोई सदस्य किसी कृषि योग्य भुमि का स्वामित्व रखता है ?

Q608 Do you or any member in your household have a bank account or a post office account? **SINGLE CODING ONLY**
क्या आप या आपके परिवार के किसी सदस्य के पास बैंक खाता या डाक घर खाता है ?

	**Q606**	**Q607**	**Q608**
Yes हाँ	1	1	1
No नही	2	2	2
	1775	1776	1777

Q609 **SHOW CARD Q609** what is the main source of drinking water for members of your household? **SINGLE CODING ONLY**
आपके परिवार के सदस्यों के लिए पीने के पानी का मुख्य स्रोत क्या है ?

Q610 **SHOW CARD Q610** what is the main source of main source of water used by members of your household for other purposes such as cooking and hand washing? **SINGLE CODING ONLY**
आपके परिवार के सदस्यों द्वारा अन्य उद्देश्यो जैसे कूकिंग और हाथ धोने के लिए इस्तेमाल किए जाने वाले पानी के मुख्य स्रोत क्या है ?

		Q609	Q610
Piped water	पाईप वाला पानी		
Piped to dwelling	घर में पाईप से	01	01
Piped to yard/plot	यार्ड/प्लॉट में पाईप से	02	02
From public tap, standpipe	पब्लिक नल/स्टैंडपाईप से	03	03
Water from tube well or borehole	नलकूप या बोरवेल से पानी	04	04
Rainwater	बरसात का पानी	05	05
Dug well	खुदे हुए कुएँ		
Protected well	सुरक्षित कुआँ	06	06
Unprotected well	असुरक्षित कुआँ	07	07
Water from spring	झरने का पानी		
Protected spring	सुरक्षित झरना	08	08
Unprotected spring	असुरक्षित झरना	09	09
Tanker truck	टेंकर ट्रक	10	10
Cart with small tank	ठेले वाला छोटा टेंक	11	11
Surface water (river/dam/ lake/pond/stream/canal/irrigation channel)	सतह का पानी (नदी/ डेम/ झील/ तालाब/भाप/नहर/सिंचाई चैनल)	12	12
Bottled water	बोतल का पानी	13	13
Other (please specify) अन्य (कृप्या स्पष्ट करे) ____________			
		1778-79	1780-81

Q611 Do you do anything to the water to make it safer to drink? **SINGLE CODING ONLY**
क्या आप पानी को सुरक्षित पीने का पानी बनाने के लिए कुछ करते है ?

Yes हाँ	1	**→ GO TO 612**
No नही	2	**→ GO TO 613**
	1782	

ASK IF '1' CODED IN Q611
Q612 **SHOW CARD Q612** Please tell me what do you do to make the water safer to drink? **SINGLE CODING ONLY**
कृप्या मुझे बताए आप पानी को सुरक्षित पीने का पानी बनाने के लिए क्या करते है ?

Boil it	उसे उबालते है	1
Filter it using water filter	वॉटर फिल्टर का उपयोग करके उसे फिल्टर करते है	2
Use water purifier/ RO	वॉटर प्युरीफायर/ आर ओ का इस्तेमाल करते है	3
Any other (please specify) ________	कोई अन्य (कृप्या स्पष्ट करे)	
		1783

Q613 Do you use a different water source for your children? **SINGLE CODING ONLY**
क्या आपके बच्चो के लिए आप अलग अलग पानी के स्रोत उपयोग करते है ?

Yes हाँ	1	**→ GO TO 614**
No नही	2	**→ GO TO 615**
	1784	

ASK IF '1' CODED IN Q613

Q614 **SHOW CARD Q614** Which water source do you use for your children? **SINGLE CODING ONLY**
आपके बच्चो के लिए आप कौन सा पानी का स्रोत इस्तेमाल करते है ?

Piped water	पाईप वाला पानी	
Piped to dwelling	घर में पाईप से	01
Piped to yard/plot	यार्ड/प्लॉट में पाईप से	02
From public tap, standpipe	पब्लिक नल/स्टैंडपाईप से	03
Water from tube well or borehole	नलकूप या बोरबेल से पानी	04
Rainwater	बरसात का पानी	05
Dug well	खुदे हुए कुएँ	
Protected well	सुरक्षित कुआँ	06
Unprotected well	असुरक्षित कुआँ	07
Water from spring	झरने का पानी	
Protected spring	सुरक्षित झरना	08
Unprotected spring	असुरक्षित झरना	09
Tanker truck	टेंकर ट्रक	10
Cart with small tank	ठेले वाला छोटा टेंक	11
Surface water (river/dam/ lake/pond/stream/canal/irrigation channel)	सतह का पानी (नदी/ डेम/ झील/ तालाब/भाप/नहर/सिंचाई चैनल)	12
Bottled water	बोतल का पानी	13
Other (please specify) अन्य (कृप्या स्पष्ट करे) ____________		

1785-86

Q615 Do you do anything to the water to make water safer for your children to drink? **SINGLE CODING ONLY**
क्या आप पानी को सुरक्षित पीने का पानी बनाने के लिए कुछ करते है ?

Yes हाँ	1	→ **GO TO 616**
No नही	2	→ **GO TO 617**

1787

ASK IF '1' CODED IN Q615

Q616 **SHOW CARD Q616** Please tell me what do you do to make the water for your children safer to drink? **SINGLE CODING ONLY**
कृप्या मुझे बताए आपके बच्चे के लिए पीने के पानी को सुरक्षित बनाने के लिए आप क्या करते है ?

Boil it	उसे उबालते है	1
Filter it using water filter	वॉटर फिल्टर का उपयोग करके उसे फिल्टर करते है	2
Use water purifier/ RO	वॉटर प्युरीफायर/ आरओ का इस्तेमाल करते है	3
Any other (please specify) _______	कोई अन्य (कृप्या स्पष्ट करे)	

1788

Q617 **SHOW CARD Q617** What type of fuel does your household mainly use for cooking? **SINGLE CODING ONLY**
आपके परिवार मे खाना पकाने के लिए मुख्य रूप से किस प्रकार का ईंधन उपयोग किया जाता है ?

Electricity	बिजली	01
LPG/ Natural Gas	एलपीजी/ प्राकृतिक गैस	02
Biogas	बायोगैस	03
Kerosene	मिट्टी का तेल	*04*
Coal/ Lignite	कोयला/ लिगनाइट	05
Charcoal	चारकोल	06
Wood	लकडी	07
Straws/ shrubs/ grass	घास/ भुसा/ तिनके	08
Agricultural crop waste	कृषि फसल का कचरा	09
Dung Cakes	गोबर के उपले	10
Any Other ____________________	कोई अन्य	

1811-12

SHOWCARD Q618

Q618 Please look at this card and tell me what kind of toilet facility do members of your household usually use? **SINGLE CODING ONLY**
कृप्या इस कार्ड को देखे और मुझे बताए आपका परिवार आमतौर पर किस प्रकार की शौचालय सुविधा का इस्तेमाल कर रहा है ?

**FLUSH OR POUR FLUSH TOILET**	फ्लश या पानी डालने वाला फ्लश टॉयलेट	
Flush to piped sewer system	पाईप सीवर सिस्टम वाला फ्लश	01

Flush to septic tank	सेप्टिक टेंक वाला फ्लश	02
Flush to pit latrine	पिट लैट्रीन वाला फ्लश	03
Flush to somewhere else	कहीं और फ्लश	04
Flush, don't know where	फ्लश कहाँ है नहीं जानता	05
**PIT LATRINE**	पिट लैट्रीन	
Ventilated improved pit (VIP) / biogas latrine	वेनटीलेटेड इम्प्रूव्ड पिट (वीआईपी)/बॉयोगैस लैट्रीन	06
Pit latrine with slab	स्लेब वाली पिट लैट्रीन	07
Pit latrine without slab/ Open pit	बिना स्लेब वाली पिट लैट्रीन/ खुला पिट/ गड्ढा	08
Twin pit/composting toilet	ट्वीन/दो पिट/ कम्पोजिटिंग टॉयलेट	09
Dry toilet	ड्राई टॉयलेट	10
No facility/uses open space Or field	कोई सुविधा नहीं/ खुली जगहों या मैदान का इस्तेमाल करते हैं	11
Any Other<br>कोई अन्य<br>__________________		

1813-14

Q619 And how many toilets are there in your household? **RECORD VERBATIM WITH LEADING ZEROES**
और आपके परिवार मे कितने शौचालय है ?

**No. of toilets in HH**<br>परिवार मे शौचालयो की संख्या		

1815-16

Q620 Do you share this toilet facility with other households? **SINGLE CODING ONLY**
क्या आप अन्य परिवारो के साथ इस टॉयलेट सुविधा को बांटते है ?

Yes हाँ	1
No नहीं	2

1817

Q621 **SHOWCARD Q621** Please look at this card and tell me, does your household or any member of your household have/own: **MULTIPLE CODING POSSIBLE**
कृप्या इस कार्ड को देखे और मुझे बताए, क्या आपके परिवार या परिवार के किसी सदस्य के पास …….. का स्वामित्व है।

Electricity Connection	बिजली का कनेक्शन	01	Motorcycle / scooter	मोटरसाइकिल/ स्कूटर	16
Mattress	मैट्रस	02	Refrigerator	रेफ्रीजरेटर	17
Pressure cooker	प्रेशर कूकर	03	Mobile phone	टेलीफोन/ मोबाइल	18
Chair	कुर्सी	04	Any other type of phone	कोई अन्य प्रकार का फोन	19
Cot or bed	कोट या बिस्तर	05	B/W Television	ब्लैक/व्हाइट टेलीविजन	20
Table	टेबल	06	Color Television	कलर टेलीविजन	21
Sewing machine	सिलाई मशीन	07	Washing Machine	वॉशिंग मशीन	22
Watch/ clock	घडी	08	CD/ VCD/ DVD player	सीडी/ वीसीडी/ डीवीडी प्लेयर	23
Bicycle	साइकिल	09	Radio or transistor	रेडियो या ट्रंसिस्टर	24
Animal drawn cart	जानवर द्वारा चलने वाली गाडी	10	Personal computer/ laptop	पर्सनल कंप्यूटर/ लैपटॉप	25
Water pump	पानी का पम्प	11	Car	कार	26
Tractor	ट्रैक्टर	12	Truck/ jeep/ van	ट्रक/ जीप/ वैन	27
Electric fan	बिजली का पंखा	13	Air conditioner	एयर कंडीश्नर	28
LPG stove	एलपीजी स्टोव	14	Threshers	थ्रेशर्स	29
Stove with electricity	बिजली के साथ स्टोव	15			

1818-67

Q622 How many rooms in this household are used for sleeping? **RECORD VERBATIM WITH LEADING ZEROES**
इस परिवार मे कितने कमरो को सोने के लिए इस्तेमाल किया जाता है ?

**No. of rooms used for sleeping in HH**<br>परिवार मे सोने के लिए इस्तेमाल होने वाले कमरो की संख्या		

1868-69

Q623 Main material of the floor. **RECORD OBSERVATION**
फर्श की मुख्य सामग्री

Natural Floor	प्राकृतिक जमीन	
Mud / clay/ earth	गीली मिट्टी/ चिकनी मिट्टी	01

Sand	रेत	02
Dung	गोबर	03
Rudimentary Floor	अल्पविकसित जमीन	
Raw Wood planks	कच्ची लकड़ी का तख्ता	04
Palm/ Bamboo	ताड़/बांस	05
Brick	ईंट	06
Stone	पत्थर	07
Finished floor	तैयार जमीन	
Parquet or polished wood	काठ–खण्ड या पॉलिश लकड़ी	08
Vinyl or asphalt	प्लास्टिक या डामर	09
Ceramic tiles	सेरामिक टाईल्स	10
Cement	सीमेंट	11
Carpet	कॉरपेट	12
Polished stone/marble/Granite	पालिश किया हुआ पत्थर/ मारबल/ ग्रेनाईट	13
Any Other<br>कोई<br>अन्य__________________________		

1870-71

Q624 Main material of the roof. **RECORD OBSERVATION**
छत की मुख्य सामग्री।

**NATURAL ROOFING**	प्राकृतिक छत	
No roof	कोई छत नहीं	01
Thatch/palm leaf/reed/grass	छप्पर/ताड़ के पत्ते/सरकण्डा/ घास	02
Mud	गीली मिट्टी	03
Sod/mud and grass mixture	घास/गीली मिट्टी और घास का मिश्रण	04
Plastic/polythene sheeting	प्लास्टिक/पॉलीथीन शीटिंग	05
**RUDIMENTARY ROOFING**	अल्पविकसित छत	
Rustic mat	साधारण चटाई	06
Palm/bamboo	ताड़/बांस	07
Raw wood planks/timber	कच्ची लकड़ी का तख्ता/ टिम्बर	08
Unburnt brick	बिना जली ईंट	09
Loosely packed stone	ढीले पैक्ड पत्थर	10
**FINISHED ROOFING**	तैयार छत	
Metal/gi (galvanized iron)	मेटल/जी आई	11
Wood	लकड़ी	12
Calamine/cement fiber	केलेमाईन/ सीमेंट फाईबर	13
Asbestos sheets	ऐस्बेस्टस शीट्स	14
Rcc/rbc/cement/concrete	आरसीसी/ आरबीसी/ सीमेंट/ कंक्रीट	15
Roofing shingles	रूफिग शिंगल्स	16
Tiles	टाईल्स	17
Slate	स्लेट	18
Burnt brick	जली ईंट	19
Any Other<br>कोई<br>अन्य__________________________		

1872-73

Q625 Main material of the walls. **RECORD OBSERVATION**
दीवारो की मुख्य सामग्री।

**NATURAL WALLS**	प्राकृतिक दीवारें	
No walls	कोई दीवारें नहीं	01
Cane/palm/trunks/bamboo	बेंत/ ताड़/ ट्रंक्स/ बांस	02
Mud	गीली मिट्टी	03
Grass/reeds/thatch	घास/ सरकण्डा/ छप्पर	04
**RUDIMENTARY WALLS**	अल्पविकसित दीवारें	
Bamboo with mud	गीली मिट्टी के साथ बांस	05
Stone with mud	गीली मिट्टी के साथ पत्थर	06
Plywood	प्लाईवुड	07
Cardboard	कार्डबोर्ड	08
Unburnt brick	बिना जली ईंट	09
Raw wood/reused wood	कच्ची लकड़ी/ फिर से इस्तेमाल की	10

	गयी लकड़ी	
**FINISHED WALLS**	तैयार दीवारें	
Cement/concrete	सीमेंट/कंक्रीट	11
Stone with lime/cement	चूना/ सीमेंट के साथ पत्थर	12
Burnt bricks	जली ईंटें	13
Cement blocks	सीमेंट ब्लोक्स	14
Wood planks/shingles	लकड़ी का तख्ता/ शिंगल्स	15
Gi/metal/asbestos sheets	जी आई/ मेटल/ ऐस्बेस्टर्स शीट्स	16
Any Other<br>कोई<br>अन्य_______________________		

1874-75

Q626 Type of Windows. **RECORD OBSERVATION**
खिडकियों के प्रकार।

		YES	NO	
Windows with glass	शीशे के साथ खिडकियां	1	2	1876
Windows with screen	स्क्रीन के साथ खिडकियां	1	2	1877
Windows with curtains / shutters	पर्दो/ शटर्स के साथ खिडकियां	1	2	1878
Any Other ___________________________	कोई अन्य	1	2	1879

SAY: I would like to know something about the **Chief Wage Earner** in your household. By Chief Wage Earner I mean the person who contributes the most to the household income.
कहेः मै आपके परिवार मे मुख्य कमाने वाले सदस्य के बारे मे कुछ जानना चाहूंगा। मुख्य कमाने वाले द्वारा मेरा मतलब व्यक्ति जो पारिवारीक आय के लिए सबसे ज्यादा योगदान देता है।

Q627 What is his/her occupation **(IF RETIRED, ASK:** What was his/her occupation before he/she retired?) **RECORD VERBATIM AND THEN CODE APPROPRIATELY IN GRID AFTER Q628.**
उनका व्यवसाय क्या है (यदि रिटायर्ड, पूछेः रिटायर होने से पहले उनका व्यवसाय क्या था?)

RECORD VERBATIM: ____

Q628 IF THE PERSON IS A BUSINESSMAN / INDUSTRIALIST, ASK "Roughly how many persons in all are employed by the CWE at his / her establishment?"
अंदाजन मुख्य कमाने वाले के व्यवसाय पर कितने लोग कार्यरत है?

**No. of employees:**				

1884-87

Q629 What is the highest level to which he/she has studied? **RECORD VERBATIM AND THEN CODE APPROPRIATELY IN GRID BELOW THEN CODE SEC).**
उनके द्वारा प्राप्त किया गया शिक्षा का उच्चतम स्तर क्या है?
RECORD VERBATIM: ____

			**Illiterate**	**School upto4yrs**	**School 5-9 yrs**	**SSC/ HSC**	**Some college but not grad.**	**Grad/Post grad.(gen)**	**Grad/ Post grad.(Prof)**	
	**Circle Education ▸**		**1**	**2**	**3**	**4**	**5**	**6**	**7**	1880
**Circle Occupation ▾**	1881-82									
**Unskilled worker**	**01**		8	8	7	6	6	6	6	
**Skilled worker**	**02**		8	7	6	5	5	4	4	
**Petty traders**	**03**		8	6	6	5	5	4	4	
**Shop owner**	**04**		6	6	5	4	3	2	2	
**Businessmen / Industrialist with no. of employees**										
**- None**	**05**		6	5	4	3	2	2	1	
**- 1-9**	**06**		5	4	4	3	2	1	1	
**- 10 +**	**07**		3	3	2	2	1	1	1	
**Self employed professional**	**08**		6	6	6	4	3	2	1	
**Clerical**	**09**		6	6	6	5	4	3	3	
**Supervisory level**	**10**		6	6	5	5	4	3	2	
**Officers / Executives**										
**- Junior**	**11**		5	5	5	4	3	2	2	
**- Middle / Senior**	**12**		3	3	3	3	2	1	1	

**SEC:**		1883

Q630 SHOWCARD Q630 Please tell me which of the following applies to your household. **SINGLE CODING ONLY**
कृपया मुझे बताये कि आपके घर पर इनमें से कौन–सा लागू होता है?

No one eat non vegetarian food	कोई भी माँसाहारी खाना नहीं खाता है	1
Some members eat non vegetarian food	कुछ सदस्य माँसाहारी खाना खाते हैं	2
All members eat non vegetarian food	सभी सदस्य माँसाहारी खाना खाते है	3

Q631 When **REFERENT CHILD** was last sick with diarrhea did you take him/ her to the doctor? **SINGLE CODING ONLY**
जब सम्बन्धित बच्चा पिछ्ली बार दस्त से बीमार था तो क्या आप उसे डॉक्टर के पास ले गयीं ?

Q632 How much did you spend for the treatment of diarrhea (consultation and medication cost)? **RECORD VERBATIM WITH LEADING ZEROES**
आपने दस्त के उपचार के लिए कितना खर्च किया (कंसलटेशन/परमर्श और दवाई के खर्च को मिलाकर) ?

	**Q631**	**Q632 (in Rs.)**				
Yes<br>हाँ	1					
No<br>नहीं	2					
Child did not suffer from this disease<br>बच्चे को यह बीमारी नहीं हुई	3					

Q633 When **REFERENT CHILD** was last sick with lower respiratory tract infection (like bronchitis, pneumonia) did you take him/ her to the doctor? **SINGLE CODING ONLY**
जब सम्बन्धित बच्चा पिछ्ली बार लोवर रेसपिटेटरी ट्रेट इनफेक्शन (जैसे फेफड़े की सूजन, नीमोनिया) से बीमार था तो क्या आप उसे डॉक्टर के पास ले गयीं ?

Q634 How much did you spend for the treatment of lower respiratory tract infection (like bronchitis, pneumonia)? **RECORD VERBATIM WITH LEADING ZEROES**
आपने लोवर रेसपिटेटरी ट्रेट इनफेक्शन (जैसे फेफड़े की सूजन, नीमोनिया) के उपचार पर कितना खर्च किया ?

	**Q633**	**Q634 (in Rs.)**				
Yes<br>हाँ	1					
No<br>नहीं	2					
Child did not suffer from this disease<br>बच्चे को यह बीमारी नहीं हुई	3					

Q503 Now, I'll read out some statements in regard with nutritional requirements of kids in the age group 6-23 months. As I read each statement, please look at this card and tell me, **SHOWCARD Q503** how strongly do you agree or disagree that this statement applies to you. **READ OUT STATEMENTS ONE BY ONE. ROTATE STATEMENTS. SINGLE CODING PER STATEMENT**
अब मै 6–23 महीनो के आयु समुह मे बच्चो की आवश्यक पोषक के संबंध मे कुछ वाक्यों को पढ़ूंगा। जैसे मै प्रत्येक वाक्य पढ़ूं, कृप्या इस कार्ड को देखे और मुझे बताए, आपके लिए यह वाक्य लागु होता इस बात से आप कितना सहमत या असहमत है।

TICK START			**Strongly disagree** पूरी तरह असहमत	**Somewhat disagree** थोडा असहमत	[illegible] **disagree** ना सहमत ना [illegible]	**Somewhat agree** थोडा सहमत	**Strongly agree** पूरी तरह सहमत	**DK/ CS (DO NOT SHOW/ READ)**	
	Carbohydrates are required to provide energy to child	बच्चे को उर्जा प्रदान करने के लिए कार्बोहाइड्रेट आवश्यक है	1	2	3	4	5	9	1632
	Calcium is important for bone development of child	बच्चे के हड्डी के विकास के लिए कैल्सियम महत्वपूर्ण है	1	2	3	4	5	9	1633
	Calcium is important for tooth development of child	बच्चे के दांतो के विकास के लिए कैल्सियम महत्वपूर्ण है	1	2	3	4	5	9	1634
	Child requires Vitamin D for proper formation of bones	हड्डियों के सही गठन के लिए बच्चे मे विटामिन डी आवश्यक है	1	2	3	4	5	9	1635
	Child requires Iron for blood development	रक्त विकास के लिए आयरन बच्चे मे आवश्यक है	1	2	3	4	5	9	1636
	Vitamin A helps in formation of healthy skin and hair of child	बच्चे की स्वस्थ त्वचा और बालो के गठन मे विटामिन ए मदद करता है	1	2	3	4	5	9	1637
	Vitamin A is good for proper eyesight of child	बच्चे की सही दृष्टि के लिए विटामिन ए अच्छा है	1	2	3	4	5	9	1638
	Child requires all nutrients for proper growth and development	बच्चे को सही विकास और वृद्धि के लिए सभी पोषको की जरूरत है	1	2	3	4	5	9	1639
	The food should have Iodine which is essential for child	खाने मे आयोडिन होना चाहिए जो कि बच्चे के लिए महत्वपूर्ण है	1	2	3	4	5	9	1640
	Fat content in food is required for physical growth of child	बच्चे के शारीरिक विकास के लिए खाने मे युक्त फैट आवश्यक है	1	2	3	4	5	9	1641
	The food should be easy to digest	खाना पचाने के लिए आसान होना चाहिए	1	2	3	4	5	9	1642
	There should be variety in food given to child so that all nutrients are provided	दिए गए आहार मे विविधता होनी चाहिए ताकि सभी पोषक प्रदान किए जा सके	1	2	3	4	5	9	1643
	Zinc is important for formation of protein in body which helps in growth of child	जिंक शरीर मे प्रोटीन की बनावट के लिए महत्वपूर्ण है जो बच्चे के विकास मे सहायक है	1	2	3	4	5	9	1644
	Child requires Zinc for blood formation	रक्त के बनने के लिए बच्चे को जिंक की जरूरत होती है	1	2	3	4	5	9	1645
	Child requires Zinc to help maintain a healthy immune system	एक स्वस्थ प्रतिरक्षा प्रणाली बनाए रखने मे मदद के लिए बच्चे को जिंक की जरूरत होती है	1	2	3	4	5	9	1646

Q508 We would like to understand what you think about infant cereals as food for your child. I will now read a list of statements and ask you if you agree with them or not. **SINGLE CODING PER STATEMENT**
हम समझना चाहेंगें आपके बच्चे के लिए खाद्य के रूप मे आप शिशु सीरियल के बारे मे क्या सोचते है। मै वाक्यों की एक सुची पढ़ूंगा और आपसे उनके साथ सहमति के लिए पूछूंगा।

TICK START			Disagree असहमत	Agree सहमत	DK/ CS (DO NOT SHOW/ READ)	
	A child that is thin or frequently sick should eat more cereals	एक बच्चा जो कि पतला है या ज्यादा बीमार होता है उसे ज्यादा अनाज चाहिए	1	2	9	1741
	Cereals are better for my child than Fast Food	अनाज मेरे बच्चे के लिए फास्ट फूड से बेहतर है	1	2	9	1742
	Homemade cereals are better for my child than packaged infant cereals	घर पर बने अनाज मेरे बच्चे के लिए पैकेज्ड अनाज से ज्यादा बेहतर है	1	2	9	1743
	Cereals contain much of the energy a child needs	अनाज मे एक बच्चे के लिए आवश्यक ज्यादा उर्जा होती है	1	2	9	1744
	Cereals contain nutrients that are lacking in other foods of children's diet	अनाज पोषक युक्त होता है जो कि बच्चे के अन्य आहार मे कम होते है	1	2	9	1745
	Cereals best suited for small children (6-23m) is different from cereals suited for children over 3 years of age and adults	३ वर्ष की आयु और बडे बच्चो के लिए उपयुक्त अनाज छोटे बच्चो के लिए उपयुक्त अनाज से अलग है	1	2	9	1746
	Cereals are required for growing children	बढते बच्चे के लिए अनाज जरूरी है	1	2	9	1747
	Cereals are required when only milk is not sufficient for child	बच्चे के लिए जब केवल दूध र्प्याप्त नही है तब अनाज जरूरी है	1	2	9	1748
	Cereals are filling	अनाज पेट भरने वाले है	1	2	9	1749

THANK & CLOSE THE INTERVIEW
